# Catalytic specificity and crystal structure of cystathionine γ-lyase from *Pseudomonas aeruginosa*

**DOI:** 10.1038/s41598-024-57625-7

**Published:** 2024-04-23

**Authors:** Marco Pedretti, Carmen Fernández-Rodríguez, Carolina Conter, Iker Oyenarte, Filippo Favretto, Adele di Matteo, Paola Dominici, Maria Petrosino, Maria Luz Martinez-Chantar, Tomas Majtan, Alessandra Astegno, Luis Alfonso Martínez-Cruz

**Affiliations:** 1 Department of Biotechnology, University of Verona, Strada Le Grazie 15, 37134 Verona, Italy; 2 Center for Cooperative Research in Biosciences (CIC bioGUNE), Basque Research and Technology Alliance (BRTA), Bizkaia Technology Park, Building 801A, 48160 Derio, Spain; 3 CNR Institute of Molecular Biology and Pathology, P.le Aldo Moro 5, 00185 Rome, Italy; 4 Department of Pharmacology, Faculty of Science and Medicine, University of Fribourg, Chemin du Musee 18, Bldg. PER17, 1700 Fribourg, FR Switzerland; 5 Centro de Investigación Biomédica en Red de Enfermedades Hepáticas y Digestivas (CIBERehd), Santander, Spain

**Keywords:** *Pseudomonas aeruginosa*, Cystathionine γ-lyase, Hydrogen sulfide, Multidrug resistant bacteria, Catalytic specificity, Crystal structure, Infectious diseases, Bacterial infection, Biochemistry, Biophysics, Structural biology, X-ray crystallography

## Abstract

The escalating drug resistance among microorganisms underscores the urgent need for innovative therapeutic strategies and a comprehensive understanding of bacteria's defense mechanisms against oxidative stress and antibiotics. Among the recently discovered barriers, the endogenous production of hydrogen sulfide (H_2_S) via the reverse transsulfuration pathway, emerges as a noteworthy factor. In this study, we have explored the catalytic capabilities and crystal structure of cystathionine γ-lyase from *Pseudomonas aeruginosa* (*Pa*CGL), a multidrug-opportunistic pathogen chiefly responsible for nosocomial infections. In addition to a canonical l-cystathionine hydrolysis, *Pa*CGL efficiently catalyzes the production of H_2_S using l-cysteine and/or l-homocysteine as alternative substrates. Comparative analysis with the human enzyme and counterparts from other pathogens revealed distinct structural features within the primary enzyme cavities. Specifically, a distinctly folded entrance loop could potentially modulate the access of substrates and/or inhibitors to the catalytic site. Our findings offer significant insights into the structural evolution of CGL enzymes across different pathogens and provide novel opportunities for developing specific inhibitors targeting *Pa*CGL.

## Introduction

The diseases caused by bacteria, fungi, or parasites pose a growing problem for today’s healthcare system. Infectious and parasitic diseases are among the top ten global causes of mortality, as identified by the World Health Organization^1^. Of particular concern is the opportunistic Gram-negative bacterium *Pseudomonas aeruginosa*, which has been designated as a critical priority for study, discovery, and the development of new antibiotics. Infections caused by *P. aeruginosa* can progress to extensive colonization and be more severe with a higher mortality rate, especially in cystic fibrosis patients and immunocompromised hospitalized individuals^2^. At present, treating *P. aeruginosa* infections effectively poses a substantial challenge owing to the bacterium’s increasing resistance to numerous clinically available antibiotics.

Recent studies have shown that a defense mechanism of bacteria against reactive oxygen species (ROS) and antibiotic-induced oxidative damage relies on the endogenous production of hydrogen sulfide (H_2_S)^3–6^. Based on these findings, inhibiting the endogenous generation of this gasotransmitter has been proposed as a strategy to combat these pathogens^3,4,7–12^, although it is still a matter of debate whether the H_2_S defensive role applies equally to all bacterial species^13,14^. Therefore, the detailed study and characterization of the enzymes involved in production of H_2_S in these organisms becomes particularly relevant for a quest to find novel avenues how to fight multidrug resistance including discovery of new antibiotics or potentiation of the current ones.

**Figure 1:**
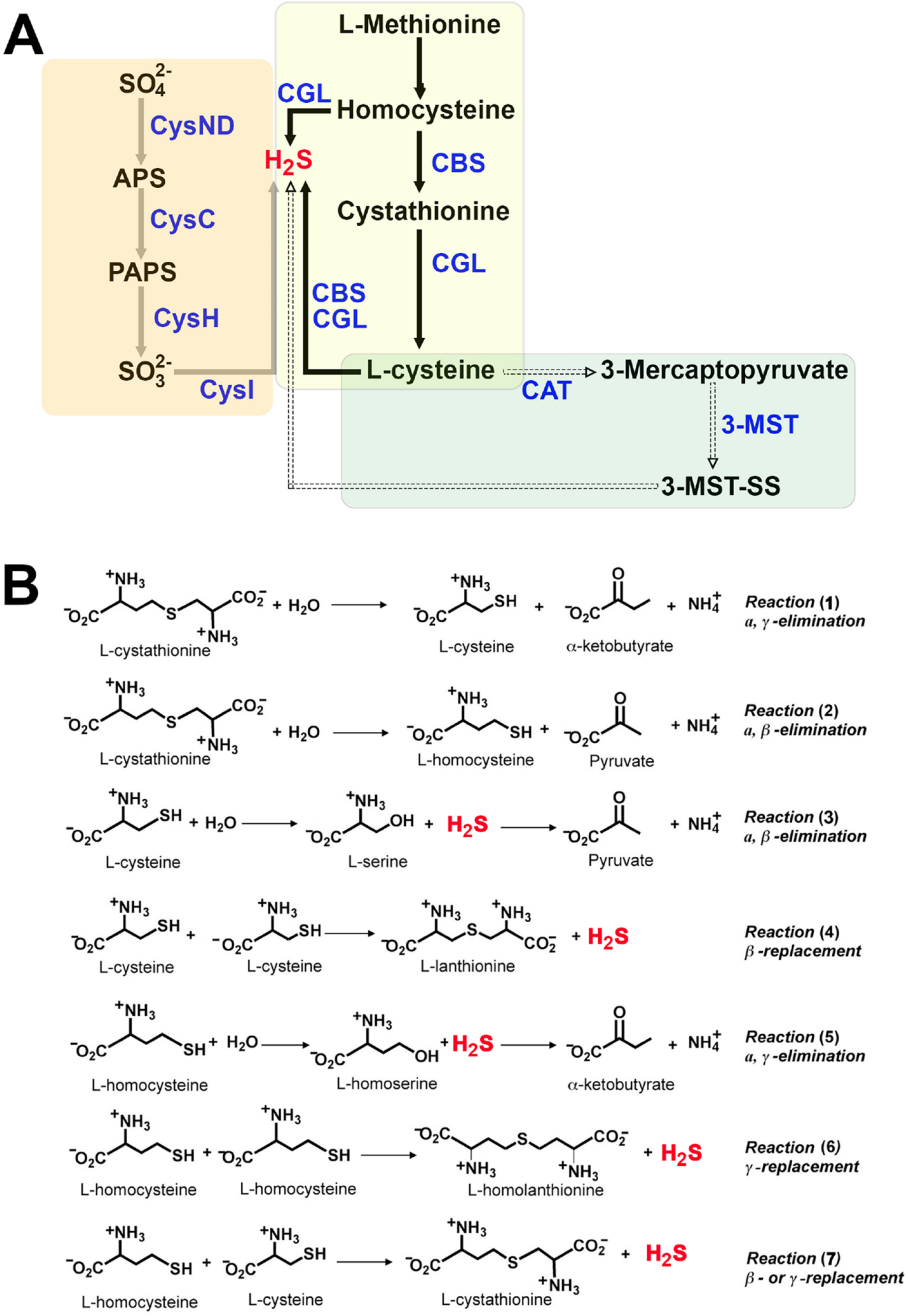
Bacterial H_2_S synthesis and reactions catalyzed by CGLs. (**A**) Scheme of the known H_2_S production pathways in bacteria: Sulfate Reduction Pathway (SRP) (grey arrows), 3-Mercaptopyruvate Sulfurtransferase (3-MST) pathway (dashed arrows) and Reverse Transsulfuration Pathway (RTP) (black arrows). The first step in the SRP is the activation of sulfate by the bifunctional sulfate adenylyltransferase subunit1/adenylylsulfate kinase (CysND), which converts ATP and sulfate to AMP and adenosine 5′-phosphosulfate (APS). The APS is transformed into 3′-phosphoadenosine 5′-phosphosulfate (PAPS) by adenylyl-sulfate kinase (CysC), and then reduced to sulfite (SO_3_^2−^) by phosphoadenosine phosphosulfate reductase (CysH). Sulfite is subsequently reduced to hydrogen sulfide (H_2_S) by sulfite reductase (SR, CysI). On the other hand, the 3-MST pathway initiates with the conversion of cysteine into 3-mercaptopyruvate by the enzyme cystine aminotransferase (CAT). CAT transfers an amino group from cysteine to α-ketoglutarate, yielding 3-mercaptopyruvate and glutamate. Once formed, 3-mercaptopyruvate serves as a substrate for the enzyme 3-Mercaptopyruvate Sulfurtransferase (3-MST). 3-MST catalyzes the transfer of a sulfur atom from 3-mercaptopyruvate to an acceptor molecule, leading to the production of H_2_S. Finally, the H_2_S production via RTP (explained detail in the “Introduction” section) involves the desulfuration of cysteine. Key enzymes in RTP are cystathionine β-synthase (CBS)^31^ and cystathionine γ-lyase (CSE) (widely described in this manuscript). Abbreviations: SO_4_^2−^ (sulfate); SO_3_^2−^ (sulfite); 3-mercaptopyruvate sulfurtransferase (3-MST); persulfidated 3-MST (3-MST-SS); Cystathionine β-synthase (CBS); Cystathionine γ-lyase (CGL); (**B**) Reactions catalyzed by CGL. The first α,γ-elimination of l-Cth (reaction **1**) represents the canonical CGL reaction, which does not lead to production of H_2_S. The α,β elimination of L-Cth (reaction **2**) refers to as the β-lyase activity of CGL which, like reaction **1**, does not produce H_2_S. However, CGL catalyzes several alternative reactions using l-Hcys and/or l-Cys as substrates generating H_2_S (reactions **3**–**7**).

The synthesis of H_2_S in bacteria varies across species and depends on substrate availability and presence of the specific enzymes. Under anaerobic conditions, the primary route for H_2_S production is the sulfate reduction pathway (SRP) involving the reduction of inorganic sulfate (SO_4_^2−^) to H_2_S^15^ (Fig. 1A). However, under aerobic conditions, the production of H_2_S by the SRP is quite inefficient compared to the specialized sulfate-reducing bacteria. Therefore, some bacteria can synthetize H_2_S through 3-mercaptopyruvate sulfurtransferase (3-MST) under conditions of sulfur limitation^3^ (Fig. 1A). Finally, the third known bacterial mechanism to produce H_2_S is the reverse transsulfuration pathway (RTP), which involves the conversion of l-homocysteine (l-Hcys) to l-cysteine (l-Cys) through two consecutive steps catalyzed by two distinct pyridoxal-5′-phosphate (PLP)-dependent enzymes, the cystathionine β-synthase (CBS) and the cystathionine γ-lyase (CGL) (Fig. 1A). CBS catalyzes a β-replacement reaction in which the hydroxyl group of l-serine (l-Ser) is replaced by l-Hcys, yielding l-cystathionine (l-Cth) and H_2_O (reviewed in^16^). Next, CGL catalyzes the α,γ-elimination of l-Cth into l-Cys, α-ketobutyrate, and ammonia (Fig. 1B, reaction **1**). In addition, many CGLs can catalyze β-elimination of l-Cth as a side reaction, producing l-Hcys, pyruvate, and ammonia (Fig. 1B, reaction **2**). This reaction is referred to as the β-lyase activity of CGL. Besides these canonical reactions, both CBS and CGL can catalyze the synthesis of H_2_S using l-Cys and l-Hcys as substrates exploiting alternative reactivity (Fig. 1B, reactions **3** to **7**).

Recent studies have revealed the presence of the *Cse* gene within *P. aeruginosa* genome encoding for the CGL (*PaCGL*) and that inactivation of the *Cse* gene leads to a reduction of H_2_S production in clinical isolates of *P. aeruginosa*^3,4^. Furthermore, the studies have demonstrated that experimental inhibitors of human CGL (*Hs*CGL) are much less effective or entirely ineffective targeting homologous bacterial CGLs^4^. This is likely due to structural and physico-chemical differences among CGLs, which could be exploited to develop new antibiotics and/or adjuvants to treat recurrent infections.

In this study we expressed, purified, and biochemically characterized *Pa*CGL as well as solved its crystal structure. We found that *Pa*CGL hydrolyzes l-Cth via γ- and β-elimination mechanisms (Fig. 1B, reactions **1**, **2**) and, in addition, catalyzes the generation of H_2_S from l-Cys or/and l-Hcys (Fig. 1B, reactions **3**–**7**). Structural comparisons to other CGL enzymes and a complementary analysis of *Pa*CGL using deep learning predictions by AlphaFold2 (AF2) revealed significant structural diversity within the main cavities of the enzymes as well as a differently organized entrance loop that may regulate the access of substrates and/or inhibitors into the PLP-containing catalytic center. These findings open new avenues for the design of selective inhibitors of *Pa*CGL and provide significant fundamental insights into the structural evolution of CGL enzymes.

## Results

### Production and biochemical properties of *Pa*CGL

*Pa*CGL was overexpressed in *E. coli* and purified to homogeneity using Ni-NTA chromatography (Fig. S1A, Fig. S8). Gel filtration of the purified *Pa*CGL showed a molecular mass of 173 kDa, suggesting that it forms tetramers, in accordance with a subunit size of ~ 44 kDa (Fig. S1B). The UV–Vis absorption spectrum of *Pa*CGL at pH 8.0 exhibited, in addition to the protein band centered at 278 nm, a peak at 425 nm that is typical of the ketoenamine tautomer of the internal aldimine (protein-bound PLP) (Fig. S1C). *Pa*CGL binds ~ 1 mol of PLP/mol of CGL subunit with a *K*_d_ value for PLP of 0.15 ± 0.01 µM, as calculated by fluorescence titrations of apo-*Pa*CGL with PLP (Fig. S1D). The apo-*Pa*CGL displayed substantially decreased thermal stability with a melting temperature (T_m_) of 57 °C compared to the PLP-loaded holo-enzyme yielding T_m_ of 68 °C (Fig. S1E).

### Enzymatic properties of *Pa*CGL enzyme

#### Canonical reactions

**Figure 2:**
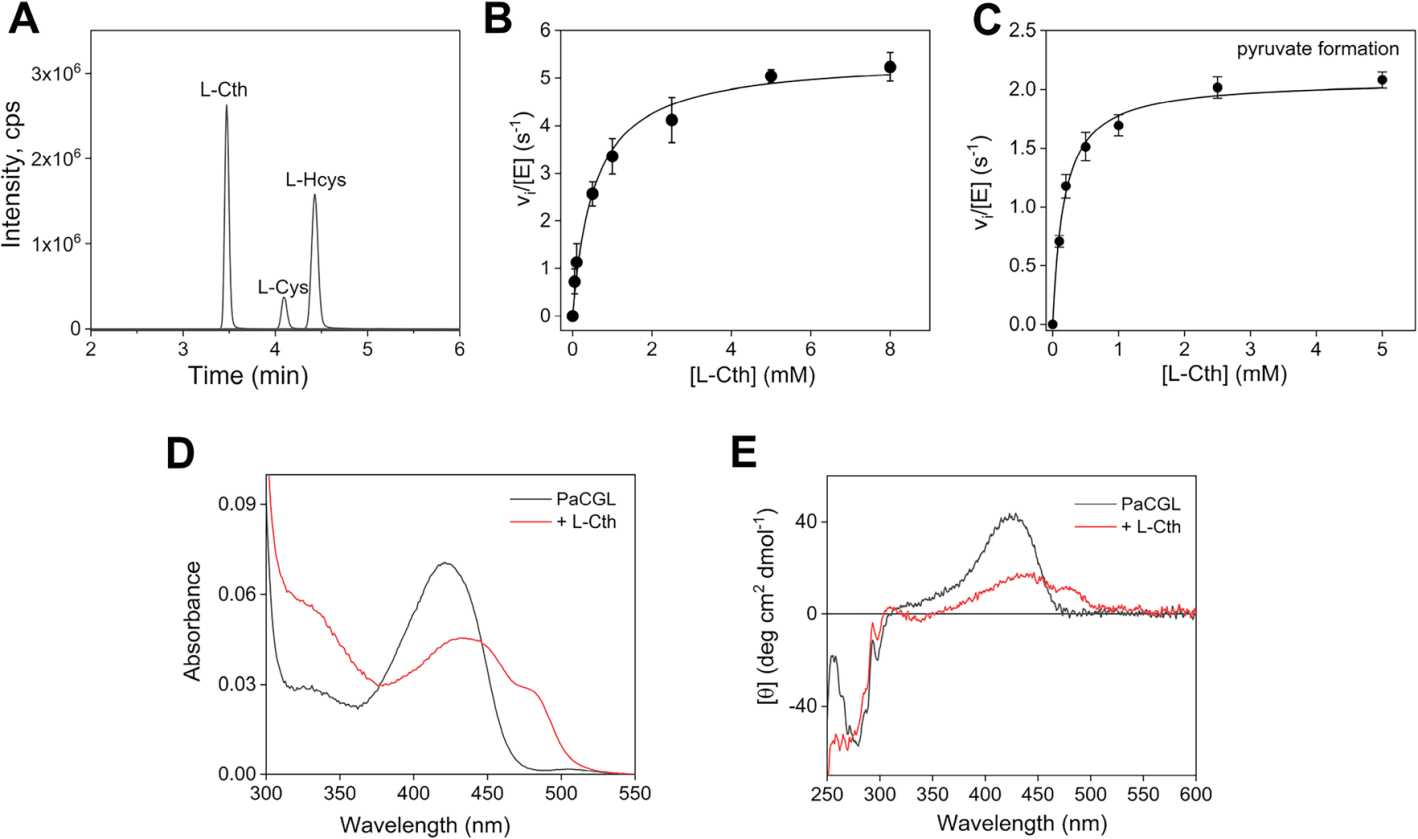
Hydrolysis of l-Cth by recombinant *Pa*CGL. (**A**) LC–MS/MS chromatogram of the amino acid products of l-Cth hydrolysis by *Pa*CGL. Parent ions with *m*/*z* values of 122 (l-Cys), 136 (l-Hcys), and 223 (l-Cth) are seen. (**B**) Steady-state initial velocity kinetics for *Pa*CGL in the hydrolysis of l-Cth measured using the DTNB assay. (**C**) Kinetics of the l-Cth hydrolysis by *Pa*CGL followed by measuring the pyruvate production via the LDH assay. Each data point in (**B**) and (**C**) represents the mean ± SEM of at least three independent experiments. (**D**) The UV–Vis absorbance spectra of 15 µM *Pa*CGL in the absence (black line) and presence of 2.5 mM l-Cth (red line). (**E**) CD spectra of 1 mg mL^−1^ *Pa*CGL in the absence (black line) and presence of 2.5 mM l-Cth (red line).

The reaction catalyzed by CGL in the transsulfuration pathway involves elimination at the γ-carbon of l-Cth. However, it is well established^17–19^ that CGLs, owing to the chemistry of the catalyzed reaction, exhibit an appreciable cystathionine β-lyase (CBL)-like activity, i.e., they can cleave both the C–γ–S and C–β–S bonds of l-Cth producing l-Cys or l-Hcys, respectively (Fig. 1B, reactions **1**,**2**). To distinguish between these two elimination reactions, the reaction products were analyzed by LC–MS/MS. As shown in Fig. 2A, when l-Cth (*m*/*z* = 223) was used as a substrate, both l-Cys (*m*/*z* = 122) and l-Hcys (*m*/*z* = 136) were observed, consistent with the enzyme’s ability to catalyze both γ-elimination (reaction **1**) and β-elimination (reaction **2**) of l-Cth.

The kinetics of l-Cth hydrolysis by *Pa*CGL was characterized using the DTNB assay. Firstly, we determined the optimal pH and temperature for *Pa*CGL activity, which were pH 8 and 42 °C, respectively (Fig. S2). However, considering the physiological temperature of 37 °C, we opted to conduct enzymatic characterization of *Pa*CGL at pH 8.0 and 37 °C. The enzymatic kinetics for the hydrolysis of l-Cth by *Pa*CGL revealed *k*_*cat*_ and K_m_ values of 5.2 ± 0.2 s^−1^ and 0.30 ± 0.04 mM, respectively (Fig. 2B, Table 1). The kinetics of pyruvate formation (reaction **2**) from l-Cth was also characterized using the LDH assay (Fig. 2C and Table 1).

**Table 1:** Kinetic parameters determined for reactions catalyzed by *Pa*CGL. Values correspond to the means ± SEMs of at least three independent experiments.

	Reaction number (Fig. 1B)	k_cat_ (s^−1^)	K_m_ (mM)	K_i_ (mM)	k_cat_/K_m_ (mM^−1^ s^−1^)
Hydrolysis of l-Cth^a^	**1 + 2**	5.2 ± 0.2	0.30 ± 0.04		17 ± 3
Pyruvate generation from l-Cth^b^	**2**	2.2 ± 0.1	0.22 ± 0.01		10 ± 1
H_2_S generation from l-Cys^c^	**3**	0.31 ± 0.02	0.43 ± 0.03	16 ± 4	0.7 ± 0.1
	**4**	0.6 ± 0.1	33 ± 6		0.02 ± 0.01
Pyruvate generation from l-Cys^b^	**3**	0.5 ± 0.1	3.2 ± 0.6	11 ± 3	0.16 ± 0.06
H_2_S generation from l-Hcys^c^	**5 + 6**	0.42 ± 0.04	1.7 ± 0.3		0.25 ± 0.07

^a^Activity was determined using the DTNB assay.

^b^Activity was determined using the LDH assay.

^c^Activity was determined using the lead acetate assay.

We also monitored the behavior of the enzyme-bound PLP in the presence of l-Cth by absorption and CD spectroscopy (Fig. 2D,E). Addition of l-Cth to *P*aCGL resulted in the pronounced absorption difference in the region of 440–480 nm with positive CD signals. For PLP-dependent enzymes involved in γ- and β-elimination reactions, absorbance bands in the region of 440–480 nm usually are assigned to the α-aminocrotonate or α-aminoacrylate intermediates^20,21^. The absorption region of 320–340 nm is not considered because pyruvate produced during the turnover also contributes to the absorption in this region.

#### Alternative reactions

**Figure 3:**
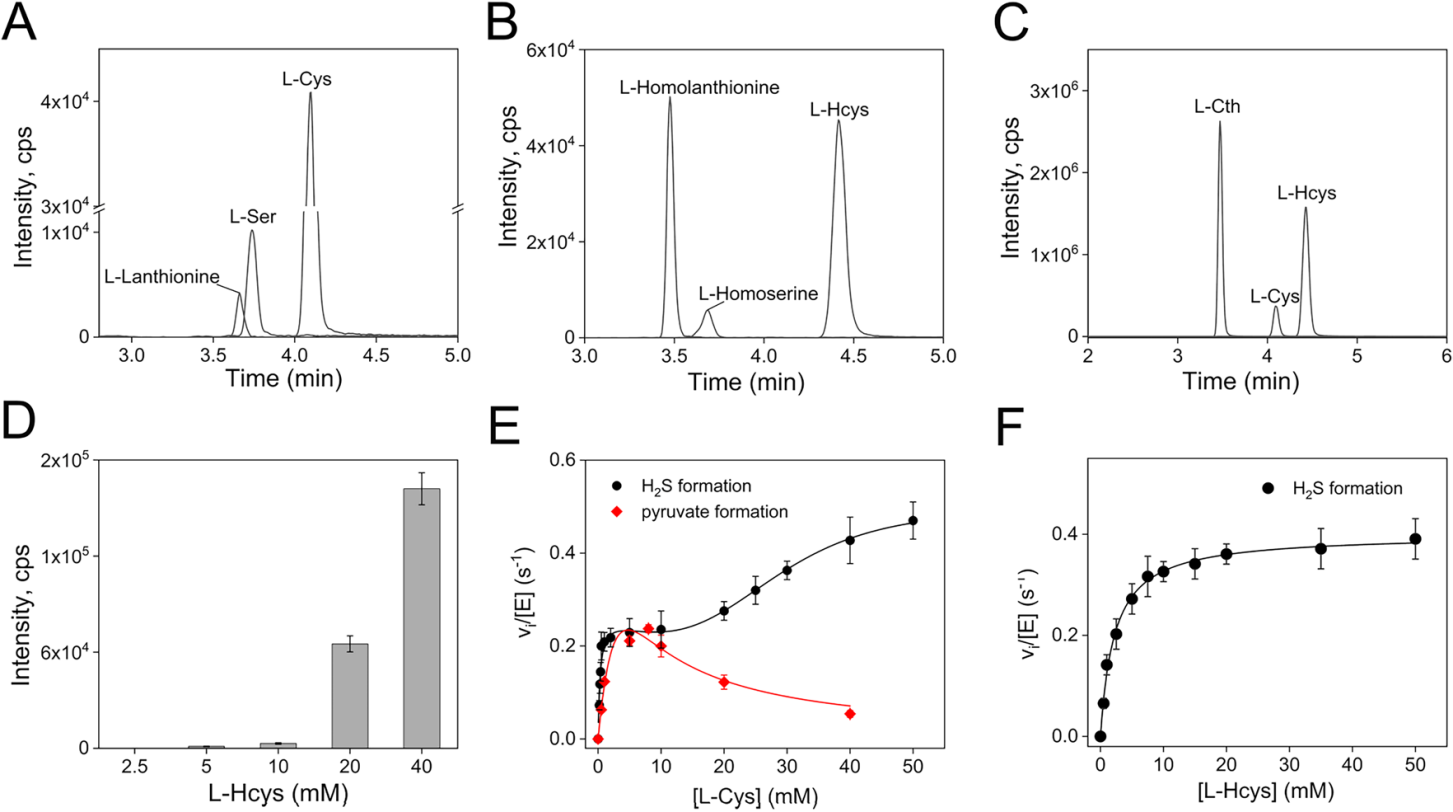
H_2_S alternative reactions. (**A**–**D**) Product analysis by LC–MS/MS of the *Pa*CGL-catalyzed reactions in the presence of l-Cys alone (**A**), l-Hcys alone (**B**) or l-Cys and l-Hcys (**C**,**D**). Parent ions with *m*/*z* values of 122 (l-Cys), 106 (l-Ser), 209 (l-Lanthionine), 136 (l-Hcys), 120 (l-Homoserine), 237 (l-Homolanthionine) and 223 (l-Cth) are seen. (**E**) Kinetics of H_2_S (reaction **3 + 4**, black line) and pyruvate (reaction **3**, red line) generation by *Pa*CGL in the presence of l-Cys. Each data point represents the mean ± SEM of at least three independent experiments. (**F**) Kinetics of H_2_S generation (reactions **5** + **6**) by *Pa*CGL in the presence of l-Hcys.

The H_2_S-producing ability of *Pa*CGL was assessed by examining the various reactions outlined in Fig. 1B (reactions **3**–**7**). The product analysis using LC–MS/MS provided direct evidence for all five potential H_2_S-generating reactions attributed to CGL. When l-Cys served as a substrate, we observed formation of both l-Ser (*m*/*z* = 106) and l-Lanthionine (*m*/*z* = 209), confirming the enzyme's ability performing both l-Cys β-lyase (reaction **3**) and l-Cys β-replacement (reaction **4**) activities, respectively (Fig. 3A). Similarly, in the presence of l-Hcys alone, l-Homoserine (*m*/*z* = 120) and l-Homolanthionine (*m*/*z* = 237) were detected, consistent with γ-elimination (reaction **5**) and γ-replacement (reaction **6**) reactions (Fig. 3B). In the presence of both l-Hcys and l-Cys, l-Cth (*m*/*z* = 223) was detected, consistent with a replacement reaction (reaction **7**) (Fig. 3C). Specifically, product analysis in the presence of 10 mM l-Cys and increasing concentration of l-Hcys confirmed that l-Cth production increases with higher l-Hcys concentrations (Fig. 3D).

Next, we determined the kinetics of H_2_S generation from l-Cys or l-Hcys. The active site pocket of CGL has binding requirements for two amino acids, since the main substrate of the enzyme l-Cth is a condensation product of l-Ser and l-Hcys. In the H_2_S-generating reactions catalyzed by *Pa*CGL (Fig. 1B, reactions **3**–**7**), either one (reaction **3** and **5**) or both (reactions **4**, **6**, and **7**) amino acid binding pockets are occupied. We analyzed the kinetic parameters associated with the single substrate reaction (i.e. ignoring H_2_O) or bimolecular reactions involving two amino acids, as outlined in the Experimental section.

The dependence of the rate of H_2_S formation on l-Cys concentration is markedly biphasic, simplifying the deconvolution of kinetic parameters related to two distinct phases corresponding to reactions **3** (β-elimination of l-Cys) and **4** (β-replacement of l-Cys). The enzyme kinetics of pyruvate (reaction **3**) and of H_2_S (reactions **3** + **4**) formation from l-Cys are shown in Fig. 3E and the resulting kinetic parameters are detailed in Table 1. Notably, *Pa*CGL exhibits significantly higher affinity for l-Cys at site 1 (0.43 ± 0.03 mM) compared to site 2 (33 ± 6 mM), with observed cooperativity in the binding of the second l-Cys molecule (n = 3 ± 1). Interestingly, like many other CGLs^19,22–24^, *Pa*CGL also displayed inhibition by l-Cys (Table 1 and Fig. 3E).

The rate of H_2_S formation from l-Hcys is not as markedly biphasic as observed for l-Cys (Fig. 3F), even though LC–MS/MS data clearly indicate that both l-Homoserine (reaction **5**) and l-Homolanthionine (reaction **6**) are produced when l-Hcys serves as a substrate (Fig. 3B). The kinetic parameters for the overall rate of H_2_S formation (reaction **5** + **6**) are reported in Table 1.

### Overall structure of *Pa*CGL

**Figure 4:**
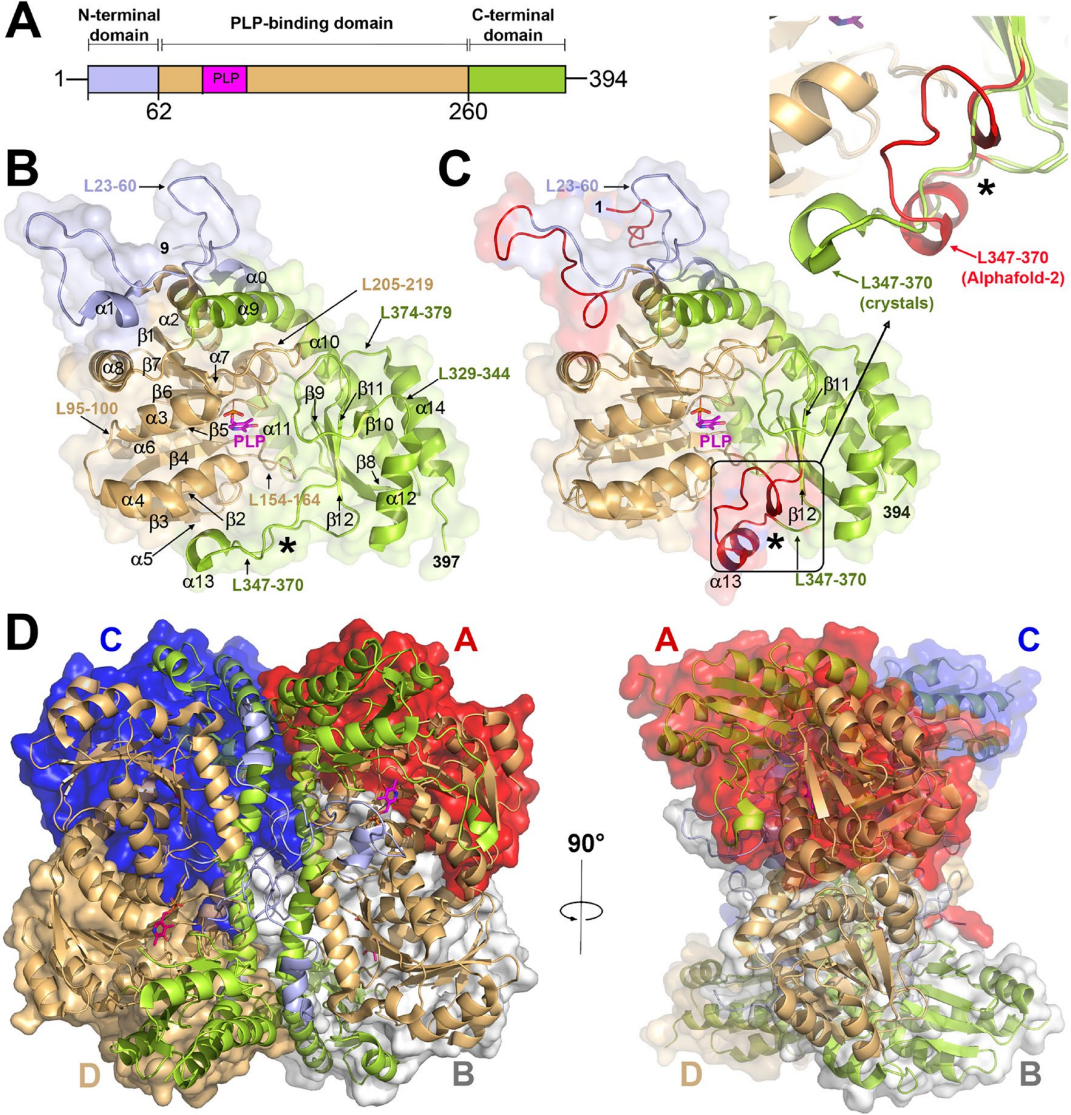
Structure of *Pa*CGL. (**A**) Domain architecture of the *Pa*CGL monomer. (**B**) Crystal structure of *Pa*CGL. The N-terminal domain (residues 1–62), the PLP-binding domain (residues 63–260), and the C-terminal module (green, residues 261–394) are colored in blue, orange, and green, respectively. PLP is in pink sticks. (**C**) Structure of *Pa*CGL predicted with AF2. Modelled amino acid residues 1–8, and 46–57 not visible in the crystals, are colored in red. The inset shows the loop L347–370 (in red, and pointed out with an asterisk), which partially defines the entrance to the catalytic cavity, and adopts a markedly different conformation in the crystals than in the predicted *Pa*CGL model. The latter is consistent with the overall structure found in other CGLs (Fig. S7). (**D**) *Pa*CGL tetrameric organization. The four subunits, A to D, are represented in different surface colors. The tetramer can be interpreted as a dimer of dimers formed by subunits A–B and C–D, respectively. The secondary elements from each subunit are colored according to the domain architecture colors shown in panel (**A**).

The crystal structure of *Pa*CGL complexed with PLP was solved at 2.0 Å (Fig. 4). The few missing or disordered segments not visible in the crystals were predicted by AF2^25^. As expected, the overall fold of *Pa*CGL is consistent with the type-I PLP-dependent enzymes, resembling human, yeast, and bacteria CGLs, as well as enzymes like cystathionine γ-synthase (CGS), or cystathionine β-lyase (CBL)^22,26^. Each *Pa*CGL subunit consists of 394 amino acids distributed in three domains: (i) the N-terminal domain (residues 1–62), (ii) the central PLP-binding domain (residues 63–260), and (iii) the C-terminal domain (residues 261–394). The N-terminal domain begins with an unstructured segment (residues 1–13), followed by a short α-helix (α0, residues 14–22) and a long loop (residues L23–60), containing a short helix α1 (residues 55–60) that is visible in only one of the four molecules of the asymmetric unit. In the remaining subunits of the tetramer, the 46–57 region of the loop appears disordered.

The PLP-binding domain is built up of a seven-stranded, mostly parallel, β-sheet (↑β1↓β7↑β6↑β5↑β4↑β2↑β3), with strand β7 (residues 219–223) antiparallel to the rest. This domain additionally contains eight α-helices, split in two sets (α1, α2, α5, α6, α7 and α3, α4, α8, respectively) that flank the central β-sheet core at both sides. The PLP-binding domain houses the catalytic center containing PLP covalently anchored to the enzyme via a conserved K208 residue and forming an internal aldimine as confirmed by the spectroscopic analysis (Fig. S1C and Fig. 2D,E). Importantly, loop L23–60 from a complementary subunit helps in defining the entrance into the catalytic cavity, and orienting PLP within the cleft. Specifically, residue R58 from the second subunit interacts with the phosphate group of the cofactor.

Finally, the C-terminal domain is organized into a five-stranded antiparallel β-sheet (exhibiting the following topology: ↑β8↓β9↑β12↓β11↓β10), decorated with five α-helices (α10–14) on one side which protect it from the solvent.

Similarly to all previously characterized CGLs, *Pa*CGL self-assembles into a homotetramer that can be described as a dimer of dimers that include subunits A–B and C–D, respectively, in which the two subunits of each dimer are related by a 2-fold axis, and the dimers themselves maintain a 2-fold symmetry (Fig. 4D). The four subunits of the tetramer exhibit high similarity (average rmsd = 0.140 Å), differing only in the conformation of two long loops (L23–60 and L347–370), which appear partially disordered or present a slightly different conformation in some of the monomers. Notably, loop L347–370 from subunit A (Fig. 4D) adopts an extended conformation in *Pa*CGL, diverging from the typical two-turn helix (α13) observed in the predicted AF2 model, and in the equivalent regions of CGLs from other species (Fig. S3A). On the other hand, loop L23–60 from the complementary B subunit of the A-B dimer was also nicely defined. This contrasts with the poor electron density observed for the region 46–57 in the other three subunits and similarly for the segment 350–366 in subunits B, C and D.

**Figure 5:**
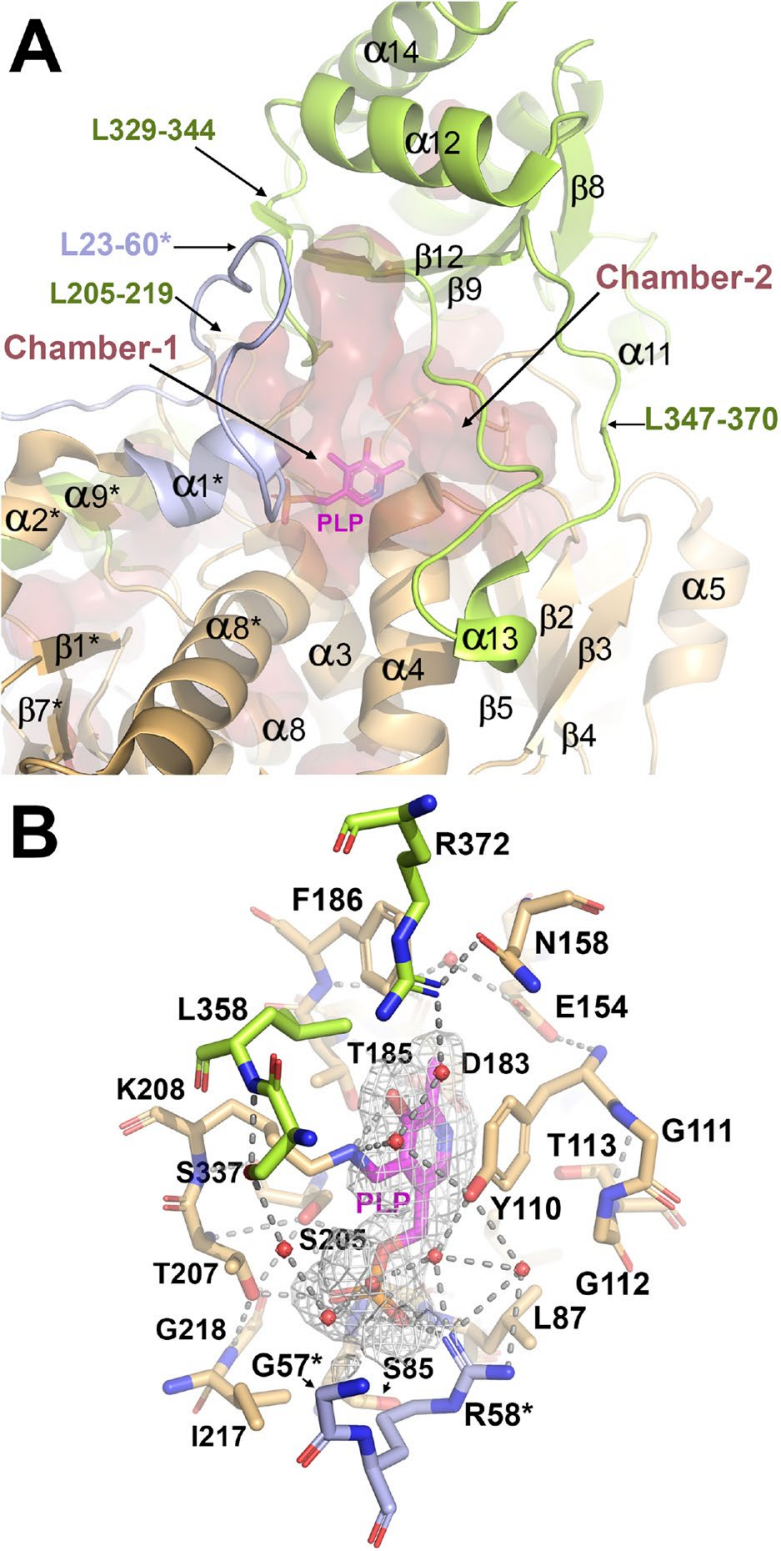
Main cavities and active site of *Pa*CGL. (**A**) Main structural elements configuring the active site. Asterisks indicate elements from a complementary subunit. PLP is in sticks. The two main cavities, chamber-1 (active site) and chamber-2 (known to host inhibitors of *Sa*CGL), are shown as red surface areas (Cavity detection cutoff = 4 solvent radii; Cavity detection radii = 7 Angstrom). (**B**) Stick representation of main amino acid residues within the PLP-binding cavity. Residues are colored according to the protein domain representation shown in Fig. 4. Polar interactions are represented in dashed lines. PLP is depicted in pink and red spheres correspond to water molecules. The Fo-Fc Polder omit electron density map (colored in light grey and contoured at 3σ) showing positive electron density around PLP is shown.

The extended conformation of the loop L347–370 directly impacts accessibility of the large cleft (chamber-2) located behind the PLP-containing catalytic site (chamber-1) (Fig. 5). Notably, the loop L347–370 does not display significant sequence conservation among various CGL homologs. Within helix α13, merely three amino acid residues remain conserved—specifically, proline, arginine, and glycine (P357, R361, G365 in *Pa*CGL)—found within the loop´s second segment (Fig. S3). Strikingly, the AF2-predicted *Pa*CGL model maintains the two-turns helicity fold present in other CGL enzymes (Fig. S3) suggesting the probability of two different (and stable) conformations for this segment. Differential conformation of this loop may modulate the accessibility of chamber-2, known to host inhibitors of *Staphylococcus aureus* CGL (*Sa*CGL)^4^ and dictate the specificity of such inhibitors to *Pa*CGL versus other species. Interestingly, the available crystal structures of various CGLs suggest a potential interdependence in the mobility of segments L23–60 and L347–370. Specifically, both loops appear either ordered or disordered simultaneously in the *Pa*CGL crystals (Fig. S4), thereby influencing the accessibility of both the catalytic cavity (loop L23–60) and the chamber-2 (loop 347–370). To decipher the differential conformation of these loops, we compared the flexibility of each subunit within *Pa*CGL tetramer with the equivalent regions in all available CGL homologs. The analysis, performed with CABSflex 2.0^27^, compared the fluctuation plots for the different protein regions and confirmed a significantly higher mobility of the L23–60 and L347–370 regions in comparison with other protein segments. Moreover, these loops also showed the highest B-thermal parameters in the crystal structures of various CGLs (Fig. S5). The heightened mobility and potentially cooperative role of a third loop comprising residues 290–297 in *Pa*CGL may potentially be involved in a novel regulatory mechanism modulating access to the chamber-2.

The active site of *Pa*CGL (Figs. 4, 5) is located within a deep cavity of each monomer situated at the dimerization interface between subunits A–B (or equivalently, C–D) (Fig. 4), whose entrance is defined by four long loops. The first three (L205–219, L329–344, and L347–370) belong to the monomer containing the PLP cofactor, while the fourth (L23–60 and, more specifically, residues 40–60) belongs to the complementary monomer of the dimer. The cavity is completed with the N-terminal ends of α-helices containing residues 85–95 (α3) and 110–126 (α4) (Fig. 4). Helix α3 dipole moment helps orienting the phosphate moiety of PLP, while the side chain of the residue Y110 located on helix α4 packs parallel to the pyridine ring of the cofactor. On the other hand, loop L205–219 provides residue K208, a conserved lysine forming an internal aldimine with PLP cofactor, thus also determining the final orientation of PLP within the cavity. As the *Pa*CGL tetramer assembly shows 222 symmetry, this particular arrangement of secondary elements is reproduced for each of the four catalytic cavities present in the CGL tetramer. Interestingly, loops L23–60 and L347–360 exhibit the highest mobility in all available CGL crystal structures to date (Fig. S4), reflecting a more pronounced fluctuation of the polypeptide chain (Fig. S5). These features suggest a potential regulatory mechanism as the conformation of these loops may limit accessibility of both the catalytic cavity and the chamber-2.

The main PLP-interacting residues, which are well-conserved across CGLs, are represented by Y110, R372, K208, S337, and S205 in *Pa*CGL (Fig. 5). Residue K208 is covalently bound to PLP through its ε-amino group forming a Schiff-bond at the C4A position of PLP. The orientation of PLP is fixed by H-bond interactions between its phosphate group and the main chain nitrogen of residues G86 and L87. The hydroxyl group of S205 and T207 also stabilizes the PLP phosphate moiety. The complementary subunit interacts with O2P and O3P of PLP via the guanidine group of R58. Most CGLs also show interactions between the PLP and the conserved tyrosine of the neighboring subunit. In *Pa*CGL, residue Y56 does not interact with the phosphate moiety of PLP, but rather establishes an H-bond with the N-terminal residue Q45. This contact is possible thanks to the flexibility provided by the residue G57. Interestingly, residue G57 of *Pa*CGL is usually substituted by a conserved serine in other CGLs. Finally, the pyridoxal ring of PLP is stacked with the phenol ring of Y110.

### Chamber-2 of *Pa*CGL as potential drug binding site

**Figure 6:**
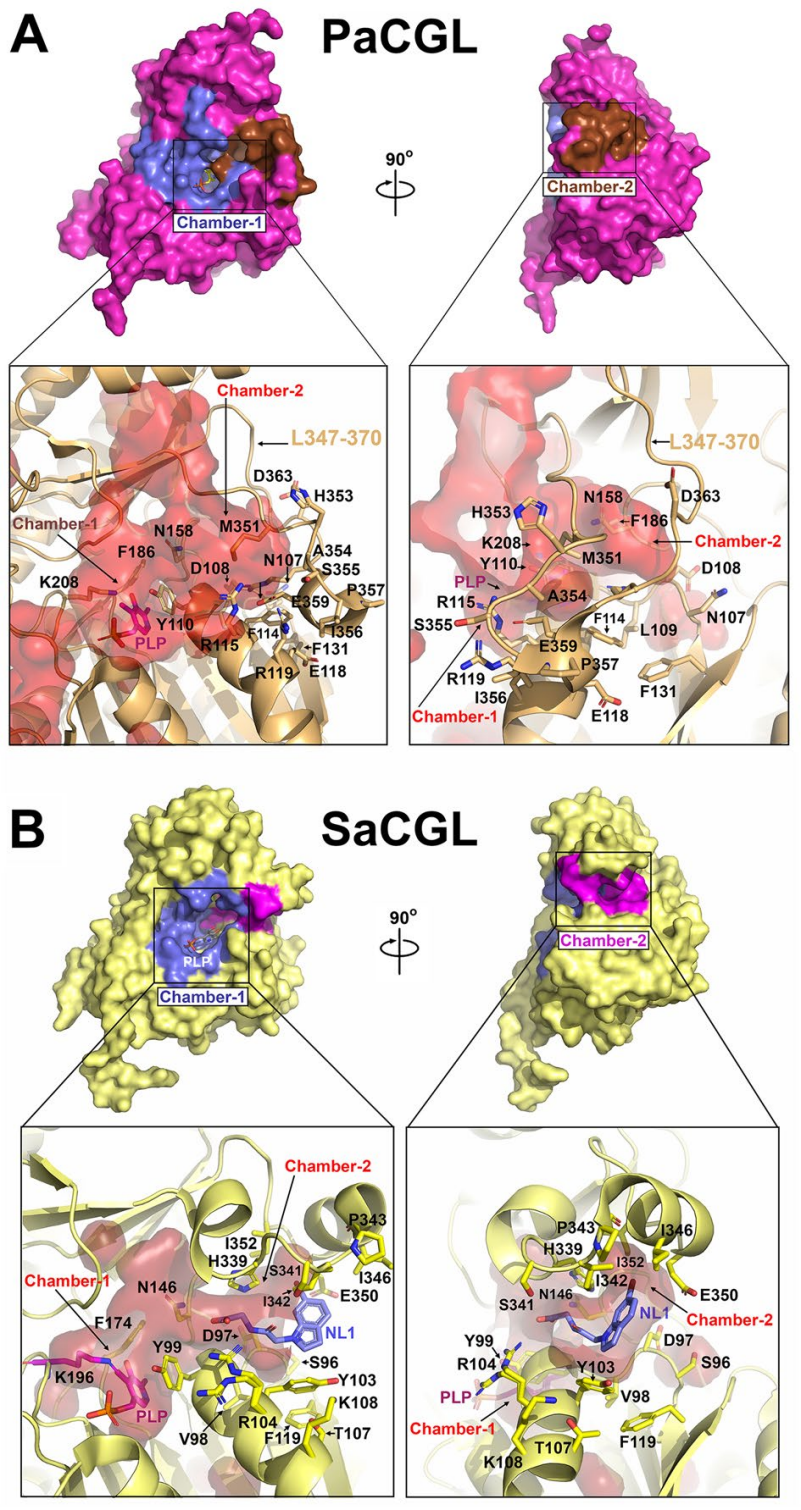
Chamber-1 and chamber-2 in *Pa*CGL and *Sa*CGL. (**A**) (Top) Surface representation of the *Pa*CGL monomer. Chamber-1 and chamber-2 are colored in blue and magenta, respectively. (Bottom) Main amino acid residues within the cavities. PLP is in sticks. (**B**) (Top) Surface representation of the *Sa*CGL monomer. Chamber-1- and chamber-2 are colored in blue and magenta, respectively. (Bottom) Main amino acid residues within the cavities. PLP is in sticks. NL1 inhibitor is in blue. The atom coordinates used to make the figure were obtained from PDB IDs 7BA4 (*Pa*CGL) and 7MCT (*Sa*CGL + NL1).

The chamber-2 deserves a detailed analysis as it has been found to host pharmacological inhibitors in *Sa*CGL^4^. Chamber-2 can be reached only via a narrow channel with limited accessibility determined by residues from the long loop connecting strands β11–β12 containing helix α13 (loop L347–370 in *Pa*CGL), and by a either tyrosine (Y103 in *Sa*CGL; Y102 in *Bc*CGL), phenylalanine (F114 in *Pa*CGL; F101 in *Lp*CGL; Fig. 6, Fig. S3) or, alternatively, asparagine residue (N137 in *Tg*CGL; N118 in *Hs*CGL). The crystal structures of *Sa*CGL complexed with novel pharmacological inhibitors NL1, NL2, and NL3 revealed the significance of the residue Y103 in *Sa*CGL catalysis and its role in stabilizing the inhibitors within the cavity through a π-stacking interaction^4^. The Y103A mutation of *Sa*CGL, or even the “humanized” Y103N variant, abolished the H_2_S-producing activity of *Sa*CGL and disrupted the interaction with the inhibitors NL1 and NL2^4^.

To assess the role of the equivalent chamber-2 residue F114 in *Pa*CGL, we replaced it with alanine or asparagine and compared the resulting variants with the wild type. The two mutations did not impair the overall structural properties as well as the tetrameric oligomerization of the purified mutant enzymes (Fig. S6A–C). Strikingly, compared to *Sa*CGL, both the F114A and the “humanized” F114N mutations resulted in only a 2–3 fold reduction in enzyme’s catalytic efficiency for both the canonical and H_2_S-producing activities of *Pa*CGL (Table 2 and Fig. S6D).

**Table 2:** Kinetic parameters for reactions catalyzed by *Pa*CGL F114A and F114N variants. Values correspond to the means ± SEMs of at least three independent experiments.

	Reaction number (Fig. 1B)	k_cat_ (s^−1^)	K_m_ (mM)	K_i_ (mM)	k_cat_/K_m_ (mM^−1^ s^−1^)
Hydrolysis of l-Cth^a^	**1 + 2**				
Wild type		5.2 ± 0.2	0.30 ± 0.04		17 ± 3
F114A		3.2 ± 0.2	0.6 ± 0.1		5.3 ± 1.2
F114N		3.2 ± 0.1	0.5 ± 0.1		6.4 ± 1.5
H_2_S generation from l-Cys^b^					
Wild type	**3**	0.31 ± 0.02	0.43 ± 0.03	16 ± 4	0.7 ± 0.1
	**4**	0.6 ± 0.1	33 ± 6		0.02 ± 0.01
F114A	**3**	0.13 ± 0.01	0.49 ± 0.01	20 ± 6	0.27 ± 0.03
	**4**	0.3 ± 0.1	33 ± 8		0.0091 ± 0.005
F114N	**3**	0.38 ± 0.06	1.6 ± 0.3	17 ± 6	0.24 ± 0.08
	**4**	0.5 ± 0.1	23 ± 3		0.02 ± 0.01

^a^Activity was determined using the DTNB assay.

^b^Activity was determined using the lead acetate assay.

Two additional amino acids situated at the base of the chamber-2 have been suggested to define the connecting gate between chamber-2 and the catalytic site (chamber-1). The first is a conserved histidine (H339 in *Sa*CGL; H356 in *Hs*CGL; H338 in *Bc*CGL; H337 in *Lp*CGL; and H376 in *Tg*CGL) (Fig. 6). *Pa*CGL also contains an equivalent histidine (H353 in *Pa*CGL), but its spatial location differs from that found in other CGLs due to the more extended conformation of the loop L347–370, and the topological position of H339 in *Sa*CGL is occupied by the M351 in *Pa*CGL structure (Fig. 6A). The second residue connecting chamber-1 and chamber-2 is a conserved tyrosine that packs against the pyridine ring of PLP and helps to orient the cofactor (Y99 in *Sa*CGL; Y114 in *Hs*CGL; Y98 in *Bc*CGL; Y97 in *Lp*CGL; Y133 in *Tg*CGL; and Y110 in *Pa*CGL, respectively). The volume and access of chamber-2 differ significantly in *Sa*CGL and *Pa*CGL (Fig. 6B). In apo-*Hs*CGL (no PLP cofactor present, PDB ID 3ELP), this tyrosine (Y114) appears displaced towards the bottom of the chamber-2 cavity, due to a partial unwinding of the last turn of helix α4. Upon binding of PLP (holo-*Hs*CGL), the last turn of this same helix recovers its helicity and reorients the tyrosine towards the interior of the catalytic chamber-1. This conformational change is thought to function as an access gate from chamber-2 to the catalytic site.

## Discussion

We conducted a biochemical and structural characterization of the CGL enzyme from *P. aeruginosa*. Our results clearly show an enzymatic competence of *Pa*CGL to generate H_2_S using alternative substrates in addition to the canonical hydrolysis of l-Cth. Detailed structural comparison of *Pa*CGL with CGL enzymes from other species revealed distinctive structural features within the primary cavities of the enzyme, which may modulate the access of substrates and/or inhibitors.

The canonical reaction of CGL within the transsulfuration pathway involves elimination of l-Cth at the γ-carbon. However, CGLs are prone to β-elimination of l-Cth as a side reaction. Our kinetic analysis demonstrated that *Pa*CGL can catalyze both the α,γ- and α,β-cleavage of l-Cth to yield l-Cys and l-Hcys, respectively. Absorbance in the region of 440–480 nm in the UV–Vis and CD spectra in the presence of l-Cth provided support for the two elimination reactions. Spectral features of the *Pa*CGL-substrate complex in the long-wavelength region are similar to those of the *Citrobacter freundii* methionine γ-lyase complex with methionine^28,29^. Two overlapping absorption bands with maxima at ~ 460 and ~ 485 nm were also observed in the yeast CGL^30^. The absorption at 480 nm likely corresponds to the formation of α-aminocrotonate, an essential intermediate of the γ-elimination reaction^30^, while absorption at around 460 nm is probably due to α-aminoacrylate formation, which has been observed under steady-state conditions for β-elimination reactions of different PLP-dependent enzymes^30–32^.

*Pa*CGL exhibited a notably high catalytic efficiency for the hydrolysis of l-Cth (17 mM^−1^ s^−1^), surpassing the activities of CGLs from other organisms (*Lb*CGL, 1.1 mM^−1^ s^−1^; *Tg*CGL, 2.2 mM^−1^ s^−1^; *Bc*CGL, 3.2 mM^−1^ s^−1^; *Sc*CGL, 2.1 mM^−1^ s^−1^; *Hs*CGL, 8.2 mM^−1^ s^−1^)^17,19,22,24,33,34^.

*Pa*CGL also produces H_2_S using l-Cys or/and l-Hcys as alternative substrates. This aligns with the previous reports showing that inactivation of the *Cse* gene, encoding for the *Pa*CGL, leads to a reduction of H_2_S production in *P. aeruginosa*^4^. We investigated various reactions catalyzed by *Pa*CGL that lead to H_2_S biogenesis and the uncommon thioether-bond-containing amino acids l-Lanthionine and l-Homolanthionine. We found that *Pa*CGL efficiently produced H_2_S via different mechanisms such as (i) *β*-elimination of l-Cys into H_2_S and l-Ser, (ii) *β*-replacement of 2 mol of l-Cys forming H_2_S and l-Lanthionine, (iii) *γ* -elimination of l-Hcys into H_2_S and l-Homoserine, (iv) *γ*-replacement of 2 mol of l-Hcys forming H_2_S and l-Homolanthionine, and (v) the replacement of l-Hcys and l-Cys producing H_2_S and l-Cth. Under substrate saturating conditions, the catalytic efficiency (*k*_cat_/*K*_*m*_) for the H_2_S elimination from l-Cys and l-Hcys is approximately 25- and 68-fold lower, respectively, than for the canonical hydrolysis of l-Cth (Table 1). However, the occurrence and regulation of these alternative reactions in the cell remain unknown. The bacterial intracellular concentrations of l-Cys are tightly regulated. Notably, *P. aeruginosa* displays a high redundancy in l-Cys production. In addition to the CGL and CBS enzymes in the RTP, this pathogen possesses genes encoding the enzymes for the de novo l-Cys synthesis pathway, i.e., serine acetyltransferase (SAT) catalyzing the condensation of l-Ser and the acetyl group of acetyl-CoA to form O-acetylserine (OAS), and the cysteine synthase (CS), which catalyzes the nucleophilic attack of sulfide (H_2_S) on OAS to form l-Cys and releasing acetate. l-Cys is also known to participate in the allosteric inhibition of SAT, leading to the production of OAS. OAS, in turn, is converted into N-acetylserine, an auto-inducer of the transcription regulator (the CysB protein), which acts as a sensor and regulator of the intracellular content of l-Cys and sulfur^35^. Further studies are required to determine whether *Pa*CGL serves as the primary checkpoint in the RTP in *P. aeruginosa* and its involvement in l-Cys production.

Shatalin et al.^4^ solved crystal structures of *Sa*CGL complexed with three novel inhibitors NL1, NL2, and NL3 demonstrating high specificity towards bacterial CGL and no impact on human (host) enzyme. Our crystal structure of *Pa*CGL enabled us to compare essential regions of this enzyme (chamber-1 and chamber-2) with the corresponding regions in other bacterial counterparts (including *S. aureus*) and the human enzyme. The analysis revealed distinct structural characteristics that set *Pa*CGL apart, potentially paving the way for future drug development targeting this important metabolic enzyme. While chamber-1, corresponding to the catalytic site, is highly conserved among CGLs and PLP-dependent enzymes in general, chamber-2 has unique physical–chemical properties and its distinct conformation sets *Pa*CGL apart from the other CGLs (Fig. 6). An intriguing difference in the chamber-2 of *Pa*CGL is the presence of the residue F114 (Fig. 6A), which occupies equivalent position of the conserved residue Y103 in *Sa*CGL (Fig. 6B). Strikingly, the F114A mutation in *Pa*CGL did not result in loss of enzyme activity, as it occurred for the equivalent Y103A mutation in *Sa*CGL, supporting the notion that this residue not only determines the general characteristics of the entrance channel to chamber-2 (volume, size, steric hindrance), but modulates the overall volume of the chamber and, consequently, the type of molecule that can be accommodated within the chamber-2. In addition, the conformation of a long loop containing helix α13 (L347–370 in *Pa*CGL) is important for accessibility of the chamber-2 (Fig. 5). This loop determines the width of the chamber-2 entrance and affects the void volume of the entire cleft (Fig. S7). Interestingly, our crystal structure of *Pa*CGL revealed that the helicity of this loop is partially lost, resulting in a more extended peptide segment that allows for a wider access into chamber-2 (a comparison of the internal cavity volume for chamber-2 and the fold of the equivalent regions to loop L347–370 in CGLs from different organisms is shown in Fig. S7). However, despite the cavity opening being bigger, the reorientation and shape of the loop L347–370 in *Pa*CGL reconfigured the internal contour and, consequently, made the volume of the cavity smaller compared to what was observed, for example, in *Sa*CGL (loop L332–356) or the human enzyme (loop L349–373) (Fig. 6 and Fig. S7). The comparison of the crystal structures of *Pa*CGL and *Sa*CGL complexed with NL1, NL2, and NL3 inhibitors suggests that the distinct arrangement of *Pa*CGL observed in this region would not hinder the binding of these molecules to *Pa*CGL, requiring only minor shifts of side chain to accommodate them inside.

The *Pa*CGL model predicted by AF2 (Fig. S7) exhibited a helical conformation of the loop L347–370 similar to that of other CGLs but different from the conformation found in our *Pa*CGL crystals. This suggests that the opening and closing of the chamber-2 cavity may differ from what was proposed based on the apo- and holo-states of the human enzyme. As demonstrated in Fig. 6, Fig. S7, and Movie S1, the increased helicity of this loop in the *Pa*CGL AF2 model corresponds to a closed conformation of the chamber-2 with a restricted accessibility and volume. The H353 residue would occupy a position like that found in other CGLs. In contrast, the extended conformation observed in the crystals made the chamber-2 larger, thus likely more accessible for small molecules, such as NL1, NL2, and NL3 inhibitors. Interestingly, in this open state, the residue M351 occupies the equivalent position to the conserved histidine in other CGLs, suggesting its role in modulating the type of molecules that can be hosted inside the cavity. Future molecular dynamics studies could provide additional new insights into conformational arrangement of the chamber-2.

Overall, our findings revealed the fundamental structural traits of the *P. aeruginosa* CGL enzyme, which has gained significant attention as a potential pharmacological target due to its role in H_2_S biogenesis in this emerging and concerning pathogen.

## Experimental section

### Protein production

Gene sequence encoding for *Pa*CGL (PAO1_PA0400) with a N-terminal 6x-His Tag was synthesized by Genscript, PCR amplified and cloned into a modified pET28a expression vector (Novagen). The F114A and F114N point mutations were introduced by site-directed mutagenesis using QuikChange II Kit (Agilent), using the primers in Table S1. All constructs were verified by DNA sequencing performed by Eurofins Genomics. The *Pa*CGL constructs were transformed into *E. coli* Rosetta (DE3) expression host cells (Novagen). Cells were grown in Luria–Bertani medium at 37 °C to a turbidity of 0.6–0.8 at 600 nm. Expression was induced with 0.5 mM IPTG for 16 h at 24 °C. Cells were harvested and resuspended in 20 mM sodium phosphate pH 8.0, 150 mM NaCl, 0.1 mM DTT containing a protease inhibitor cocktail EDTA free. After sonication, the suspension was centrifuged at 30,000×*g* for 20 min at 4 °C. The supernatant was recovered and loaded on an Ni–NTA Sepharose column (GE-Healthcare) equilibrated with 20 mM sodium phosphate pH 8.0, 150 mM NaCl, 0.1 mM DTT and 10 mM imidazole. A linear gradient from 10 to 500 mM imidazole was then applied. Fractions enriched in *Pa*CGL were pooled together, concentrated and buffer exchanged into 20 mM sodium phosphate pH 8.0, 150 mM NaCl, 0.1 mM DTT buffer using Vivaspin concentrators (Sartorius). Each purification yielded about 100 mg of pure protein per liter of bacterial culture. To facilitate crystallization, an additional construct for *Pa*CGL wild-type with the C-terminal 6x-His tag was prepared using similar strategy as described above.

The theoretical extinction coefficient of monomeric *Pa*CGL at 280 nm was 28,545 M^−1^ cm^−1^ (http://www.expasy.ch/tools/protparam.html). The PLP content of the enzyme was determined by releasing the coenzyme in 0.1 M NaOH and by using ε_M_ = 6600 M^−1^ cm^−1^ at 388 nm.

The oligomeric state of *Pa*CGL variants was determined by gel filtration using a Sephacryl S-200 16/60 HR column in 20 mM sodium phosphate pH 8.0, 150 mM NaCl and 0.1 mM DTT. The calibration curve was generated following the protocols in^36,37^.

The apo-form of *Pa*CGL was obtained by incubating the enzyme with phenylhydrazine hydrochloride following the protocol in^38^. The dissociation constant for PLP (*K*d) was obtained by monitoring the change of intrinsic fluorescence (excitation was set at 295 nm) of 1 μM apo-protein at different concentrations of PLP (0.01–4 μM) in 20 mM sodium phosphate pH 8.0 at 25 °C on a FP8200 Jasco spectrofluorimeter^24,39^.

### Spectroscopic measurements

Absorption spectra of 15 μM *Pa*CGL were collected on a Jasco V-750 UV–visible spectrophotometer in 20 mM sodium phosphate pH 8.0 at 25 °C^31^. CD spectra were recorded on CD spectropolarimeter Jasco J-1500 equipped with a Peltier type thermostated cell holder, as previously described^31,40^. Briefly, far-UV (190–250 nm) spectra of 0.2 mg mL^−1^ *Pa*CGL variants were collected in using a 0.1-cm path length quartz cuvette. Near UV–Vis (250–600 nm) spectra of 1 mg mL^−1^ *Pa*CGL variants were recorded in 1-cm path length quartz cuvette at 25 °C. A minimum of three accumulations were made for each scan, averaged, and corrected for the blank solution of corresponding buffer. Thermal unfolding profiles were collected by recording ellipticity at 222 nm in a temperature range between 15 to 90 °C (scan rate 90 °C/h) using 0.1-cm path length quartz cuvettes and protein concentration of 0.2 mg mL^−1^. All CD measurements were recorded in 20 mM sodium phosphate pH 8.0^41^.

### Enzyme activity assays

The CGL activity in the l-Cth γ-elimination reaction was determined by a previously described 5,5′-dithiobis-2-nitrobenzoic acid (DTNB) assay^24,42^.

Briefly, reactions (200 µL sample) were carried out at 37 °C in assay buffer (50 mM MOPS, 50 mM bicine, 50 mM proline pH 8.0, 20 μM PLP) containing 0.2 mM DTNB in the presence of different concentrations of l-Cth (0–8 mM). The reactions were initiated by the addition of *Pa*CGL to 1 μM. Absorbance changes were monitored in continuous at 412 nm (Δε_412_ = 13,600 M^−1^ cm^−1^).

The activities in the H_2_S-generating alternative reactions were measured using the lead acetate assay as described elsewhere^31,43–45^. The enzyme (1–4 µM) was added to 0.4 mL of reaction mixture containing 50 mM HEPES pH 7.4, 20 μM PLP, 0.4 mM lead (II) acetate, and 0–50 mM l-Cys or 0–50 mM l-Hcys.

Pyruvate formation was measured by monitoring NADH oxidation (ε_340_ = 6200 M^−1^ cm^−1^) via LDH assay^36^. Data for H_2_S production from l-Cys by *Pa*CGL were fitted following the kinetic models described previously^19,45^. Briefly, H_2_S production from l-Cys is the sum of two possible reactions, the β-elimination of l-Cys to generate l-Ser (reaction **3**) or the condensation of two molecules of l-Cys to generate l-Lanthionine (reaction **4**). Data for overall H_2_S production from L-Cys were fitted using Eq. (1) where v_l-ser_ and v_Lanthionine_ are defined by Eq. (2) and Eq. (3), respectively ^19,31,45^.

1 $${v}_{H2S}={v}_{L-Ser}+ {v}_{Lanthionine}$$

2 $${v}_{L-Ser}=\frac{{v}_{max1 }[L-Cys]}{{K}_{m1(L-Cys)}+[L-Cys](1+\frac{\left[L-Cys\right]}{{K}_{i}})}$$

3 $${v}_{Lanthionine}=\frac{{v}_{max2 [L-Cys]{[L-Cys]}^{n}}}{\left[L-Cys\right]{\left[L-Cys\right]}^{n}+{K}_{m1 }{[L-Cys]}^{n}+\left[L-Cys\right]{K}_{m2}^{n}}$$

where K_m1_ and V_max1_ are associated to the unimolecular reaction, K_m2_ and V_max2_ to substrate binding at the second site and the reaction velocity of the bimolecular reaction and n represents Hill coefficient.

### Liquid chromatography mass spectrometry (LC–MS/MS)

A TSQ Fortis Triple Quadrupole mass spectrometer (Thermo Scientific) coupled to Ultimate 3000 HPLC system (Thermo Scientific) was used for this analysis. The products separation was performed on a Luna C18(2) column (150 × 4.6 mm, 3 µm particle size, Phenomenex) with gradient elution. The mobile phase was composed of formic acid (A, 0.1% formic acid in water) and acetonitrile (B, 0.1% formic acid in ACN). Chromatographic gradient elution was the following: constant flow of 0.4 mL min^−1^; 98% phase A at time 0, then decreased up to 5% A in 10 min and maintained at 5% A for 2 min and re-equilibrated for 5 min. The ESI source settings were ion spray voltage, +3500 V; ion transfer tube, 300 °C; sheath gas and aux gas, 50 and 10, respectively, vaporizer temperature 350 °C. Multiple reaction monitoring was optimized using nitrogen as collision gas (with pressure set at 1.5 mTorr). Two transitions for each substance were chosen for identification. Data acquisition and elaboration were performed by the Chromeleon (version 7.2, Thermo Fisher).

### Protein crystallization

For crystallization, the enzymes were buffer exchanged into 50 mM HEPES, 150 mM NaCl, 0.1 mM DTT pH 8.0. Preliminary crystallization trials were carried out by the vapor-diffusion technique in a sitting drop format with 96-well MRC crystallization plates, following a previously described protocol^22^.

Drops consisted of 200 nL protein solution (20 mg mL^−1^) were mixed with 200 nL precipitant solution and incubated at 293 K. The successful condition was scaled-up in a hanging-drop format using 24-well VDX plates (Hampton Research) in a reservoir with drops consisting of 0.5 μL protein (protein concentration of 20 mg mL^−1^) with 0.5 μL precipitant solution. This reservoir was composed of 9% w/v PEG 4000 and 0.1 M sodium acetate pH 4.6 with a volume of 0.5 mL. The crystals were transferred to a crystallization buffer containing 9% (w/v) PEG 4000, 0.1 M sodium acetate pH 4.6, and 20% glycerol for a few seconds before being cryocooled in liquid nitrogen.

### Structural determination by X-ray crystallography

All X-rays datasets were collected at Synchrotron beamlines XALOC (ALBA), I03/I24 (DIAMOND, UK) and ID29 of ESRF (Grenoble). Datasets were collected over a range of 0.1–0.25° and the distance to the detector was set to reach resolution data between 1.6–3.8 Å depending on the crystal, and according to the diffraction parameters previously determined by several test images. Several data set were collected but only one allowed the structural determination of *Pa*CGL (Table S2). Diffraction data were processed using HKL2000^46^ or XDS^47^ programs. The three-dimensional structure of *Pa*CGL was determined by MR method with the Phaser-MR program^48^ from Phenix Suite^49^ using the coordinates of *Hs*CGL holoenzyme (PDB ID 2NMP) as initial search model. The geometric quality of the models was assessed with MolProbity^50^ integrated in Phenix suite. Figures were done with Pymol (The PyMOL Molecular Graphics System, Version 2.2.3, Schrödinger, LLC)^51^ and UCSF Chimera^52^.

### Deep learning structural comparison

Protein structure predictions were performed with AlphaFold 2.3.0^25^ using an adapted version of the AF2 code (https://github.com/deepmind/alphafold).

### Supplementary Information

Supplementary Information.

Supplementary Legends.

Supplementary Video 1.

## Data Availability

All data generated or analysed during this study are included in this published article and its supplementary information files. The nucleotide sequence of *Pa*CGL (PAO1_PA0400) can be accessed in the *Pseudomonas aeruginosa* database (https://www.pseudomonas.com/feature/show?id=103537). Additionally, the crystal structure of *Pa*CGL (PDB code: 7BA4) is accessible through the Protein Data Bank (https://www.rcsb.org/structure/7BA4).
