# Supplementary material for: Catalytic specificity and crystal structure of cystathionine γ-lyase from Pseudomonas aeruginosa
Source: Sci Rep. 2024 Apr 23;14:9364. doi: 10.1038/s41598-024-57625-7 (PMC11039470; doi:10.1038/s41598-024-57625-7)
Supplement: Supplementary file 1 — Supplementary Information. [file 41598_2024_57625_MOESM1_ESM.pdf]

SUPPLEMENTARY INFORMATION

Catalytic specificity and crystal structure of cystathionine γ-lyase from *Pseudomonas aeruginosa*

Marco Pedretti[1, #], Carmen Fernández-Rodríguez[2, #], Carolina Conter[1,2], Iker Oyenarte[2], Filippo Favretto[1], Adele di Matteo[3], Paola Dominici[1], Maria Petrosino[4], Maria Luz Martinez-Chantar[2,5], Tomas Majtan[4], Alessandra Astegno[1, *] and Luis Alfonso Martínez-Cruz[2, *]

[1]*Department of Biotechnology, University of Verona, Strada Le Grazie 15, 37134 Verona, Italy.*
[2]*Center for Cooperative Research in Biosciences (CIC bioGUNE), Basque Research and Technology Alliance (BRTA), Bizkaia Technology Park, Building 801A, 48160 Derio, Spain*
[3] CNR Institute of Molecular Biology and Pathology, P.le Aldo Moro 5, 00185 Rome, Italy
[4]*University of Fribourg, Department of Pharmacology, Faculty of Science and Medicine, Chemin du Musee 18, Bldg. PER17, CH-1700 Fribourg, FR, Switzerland.*
[5]*Centro de Investigación Biomédica en Red de Enfermedades Hepáticas y Digestivas (CIBERehd)*

[#] These authors contributed equally to this work.
*Corresponding authors. Address: Luis Alfonso Martínez-Cruz: email: amartinez@cicbiogune.es; Tel: +34 944061318, fax: +34 944-061-301. Alessandra Astegno: email: alessandra.astegno@univr.it; Tel: +39 045-8027955, fax: +39 045-8027929

Table S1. List of mutagenic primers used to generate *Pa*CGL variants. PF = Primer Forward, PR = Primer Reverse.

Mutation	Type of primer	Primer sequence
F114A	PF	CTACGGCGGTACC**GCC**CGCCTGTTCGAGCG
	PR	CGCTCGAACAGGCG**GGC**GGTACCGCCGTAG
F114N	PF	CTACGGCGGTACC**AAC**CGCCTGTTCGAGCG
	PR	CGCTCGAACAGGCG**GTT**GGTACCGCCGTAG

Table S2 Statistics for data collection and refinement

**PDB code**	7BA4
**Beamline**	ALBA MX XALOC-BL13
**Wavelength**	1.1074
**No images/ΔΦ (°/image)/Exposure Time (sec)**	3600/0.1/0.5
**Detector**	Dectris PILATUS 6M
**Space group**	$P3_121$
**Unit cell (Å)** (a, b, c)	79.30 79.30 446.67
**Resolution (Å)**(a, b, c)	54.45 – 1.99 (2.03 - 1.99)
**CC ½ (%)**	99.50 (77.50)
**Redundancy**	18.30 (10.60)
**Completeness (%)**	99.90 (98.80)
**I/σ (I)**	13.90 (2.90)
**Wilson B-factor (Å^2)**	30.70
$\mathbf{R_{meas}}^b$	0.162 (0.820)
$\mathbf{R_{pim}}^c$	0.037 (0.247)
**Refinement**	
Total reflections	2060939 (58513)
Unique reflections	112386 (5512)
$Rwork^d/Rfree^e$	0.160/0.205
**N° of non-hydrogen atoms**	
Macromolecules	12573
Ligand (PLP)	15
**Average B-factor (Å^2)**	
Macromolecules	40.00
Ligands	37.19
**Ramachandran plot statistics (%)**	
Res. in most favored regions	98.07
In additional allowed regions	1.72
In disallowed regions	0.21
**RMSDs**	
Bonds length (Å)/ angle (°)	0.010/0.99
**Rotamers outliers (%)**	0.00
**Clashcore**	4.32
**MolProbity score** ^{f}	1.21

One crystal was used for each data set. Values in parentheses are for highest-resolution shell. Rmerge a = Σhkl Σi |Ii (hkl) − <I(hkl)>I/Σhkl Σi Ii(hkl) (all I+ and I-); ^{b}Rmeas = Σhkl Σi |Ii (hkl) − <I(hkl)>I/Σhkl Σi Ii(hkl) (all I+ and I-); ^{c} Rpim= Σhkl Σi |Ii (hkl) − <I(hkl)>I/Σhkl Σi Ii(hkl) (all I+ and I-); ^{d}Rwork = Σ |Fo – Fc | / ΣFo; ^{e} Rfree = Σ |Fo – Fc | / ΣFo, calculated using a random 5 % of reflections that were not included throughout refinement. ^{f} MolProbity score: 0.426 *ln(1+clashscore) + 0.33 *ln(1+max(0, rota_out|-1)) + 0.25 *ln(1+max(0, rama_iffy|-2)) + 0.5

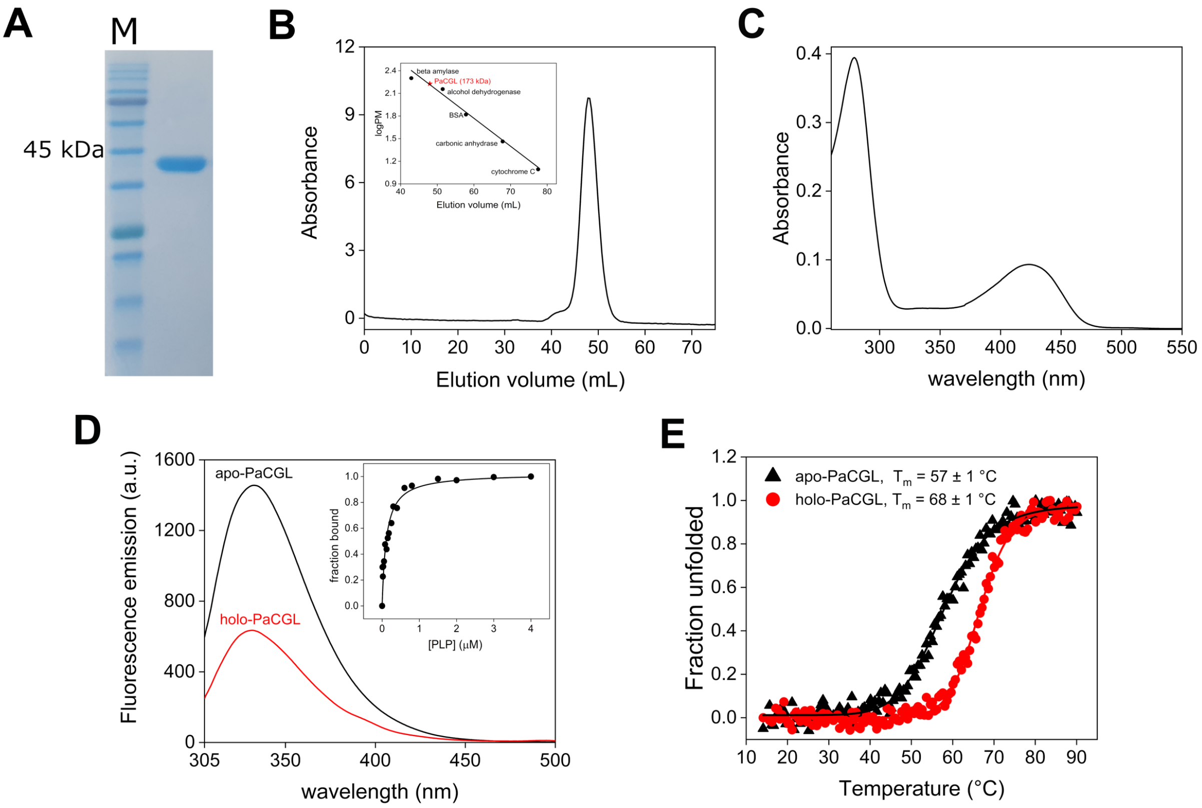

Figure S1. Properties of recombinant *Pa*CGL. (**A**) Cropped 12% SDS-PAGE gel of purified recombinant wild type *Pa*CGL (original gel is presented in Supplementary Figure S8). Lane M, protein marker. (**B**) Size exclusion chromatography of *Pa*CGL using Sephacryl S-200 16/60 high resolution column in 20 mM sodium phosphate pH 8.0, 150 mM NaCl, 0.1 mM DTT. (Inset) Calibration curve of molecular weight logarithm versus elution volumes. (**C**) UV-visible absorption spectrum of 15 μM purified PaCGL in 20 mM sodium phosphate buffer pH 8.0. (**D**) Emission spectra of 1 μM apo-*Pa*CGL before and after reconstitution with PLP in 20 mM sodium phosphate buffer pH 8. Inset, representative fluorescence titration of apo-*Pa*CGL (1 μM) with PLP (0.01– 4 μM) monitoring the quenching of intrinsic fluorescence emission at 333 nm upon excitation of the apo- *Pa*CGL at 295 nm. The K_d value is determined by fitting the fraction of bound PLP (fb) to a hyperbolic equation and represents a mean value ± SEM of three independent measurements. (**E**) Thermal denaturation profile of 0.2 mg/mL apo- (black) and holo-*Pa*CGL (red) recorded following the ellipticity signal at 222 nm in 20 mM sodium phosphate buffer pH 8.0.

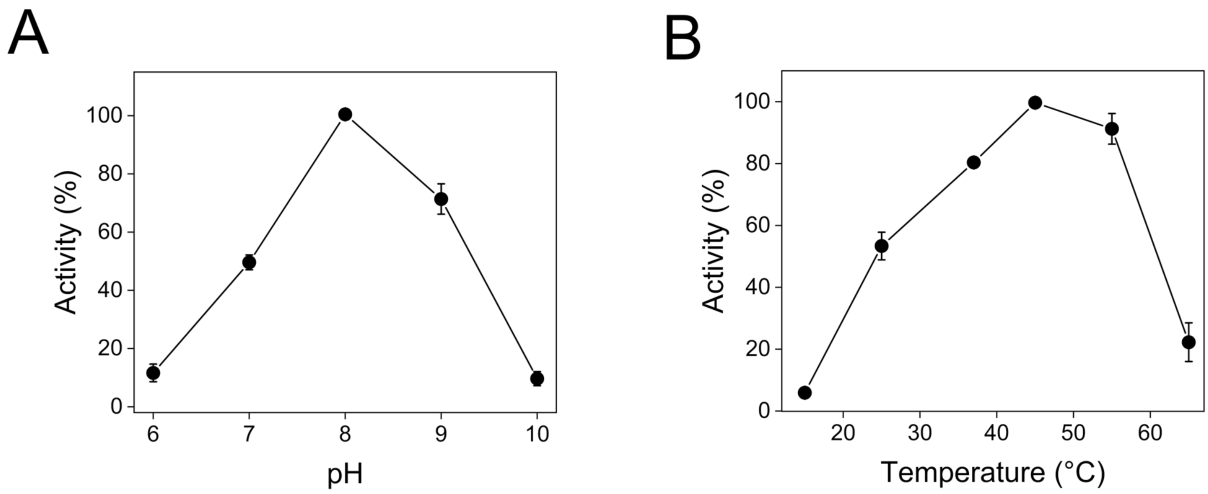

Figure S2. Effect of pH and temperature on *Pa*CGL γ-elimination of L-Cth. (A) pH-dependent activity profile for *Pa*CGL performed at constant saturating L-Cth concentration in the pH range of 6-10 at 37°C. **(B)** *Pa*CGL enzyme activity over temperature range of 15-65°C at constant saturating L-Cth concentrations at pH 8.0. Data points correspond to average values of four independent measurements, while error bars represent SEMs.

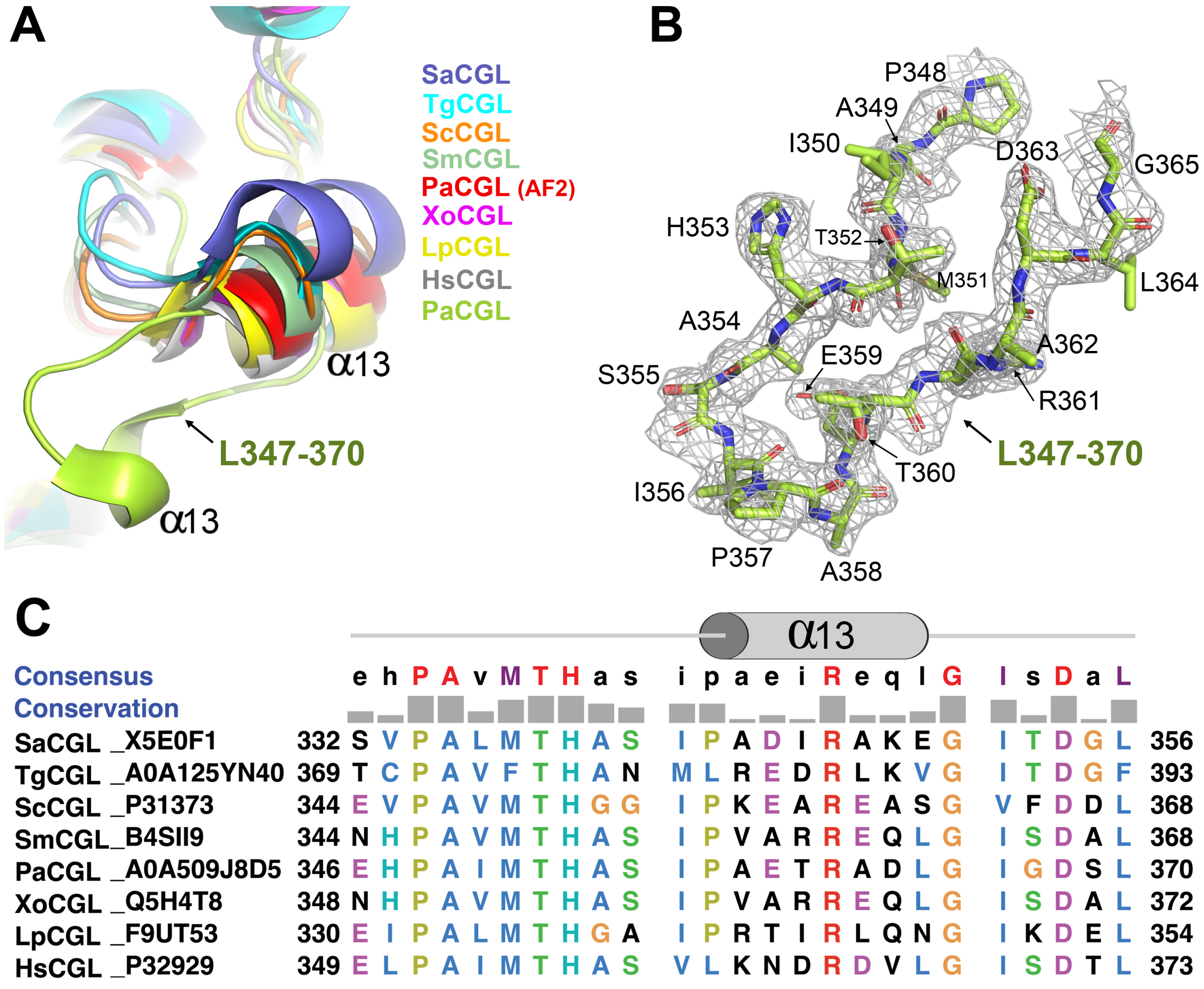

Figure S3. Conformation of the loop 347-370 in *Pa*CGL and homologs. (**A**) Zoomed view of the loop L347-370 in *Pa*CGL and homolog enzymes obtained from the crystal structure of CGLs from various species, and from the Alphafold-2 predicted model of *Pa*CGL. *Sa*CGL, *Staphylococcus aureus* (PDB ID 7MCB); *Tg*CGL, *Toxoplasma gondii* (PDB ID 7NL1); *Sc*CGL, *Saccharomyces cerevisiae* (PDB ID 1N8P); *Sm*CGL, *Stenotrophomonas maltophila* (PDB ID 6K1L); *Xo*CGL, *Xanthomonas oryzae* (PDB ID 4IYO); *Lp*CGL, *Lactobacillus plantarum* (PDB ID 6LDO); *Hs*CGL, Homo sapiens (PDB ID 2NMP); *Pa*CGL, *Pseudomonas aeruginosa*-crystal structure (PDB ID 7BA4); *Pa*CGL-AF2 (*Pseudomonas aeruginosa*, predicted with Alphafold-2). (**B**) Detailed view of the loop L347-370 of *Pa*CGL observed in the crystals and 2FoFc σ-weighted electron density map, contoured at 1 σ. (**C**) Alignment of the amino acid sequence from loop 347-370 of *Pa*CGL with the homologous region of the organisms depicted in panel A. The numbering of the corresponding amino acids and the UniProt code of each protein are indicated in the figure. The upper part displays the consensus sequence and the level of conservation resulting from the alignment using Clustal Omega (https://www.ebi.ac.uk/Tools/msa/clustalo/). Residues included in the commonly observed two-turn helix α13 are indicated with a cylinder.

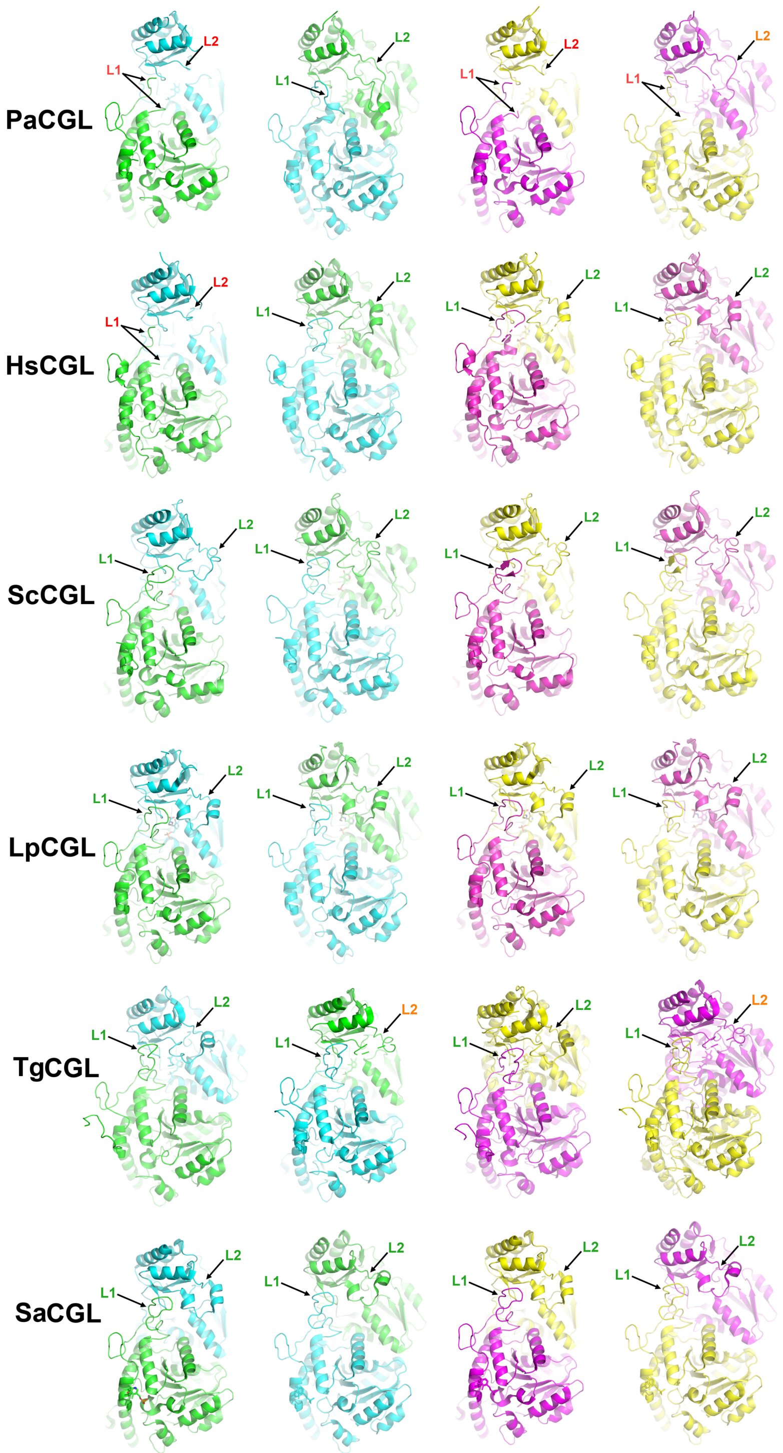

Figure S4. Structural features of loops L23-60 and L347-370 in different CGLs. Loop L23-60 and L347-370 and their homologs, indicated by arrows, are depicted as L1 and L2, respectively. Green, orange and red letters denote ordered, partially disordered, and mostly disordered loops, respectively. Of note, the order/disorder of loop L1 affects concomitantly the order/disorder of loop L2 and vice versa. The depicted CGLs are as follows: *Pseudomonas aeruginosa* (*Pa*) - PDB code: 7BA4; *Homo sapiens* (*Hs*) - PDB code: 2NMP; *Saccaromyces cerevisiae* (*Sc*) - PDB code: 1N8P; *Lactobacillus plantarum* (*Lp*) - PDB code: 6LE4; *Toxoplasma gondii* (*Tg*) - PDB code: 8BIZ; *Staphylococcus aureus* (*Sa*) - PDB code: 7MCL.

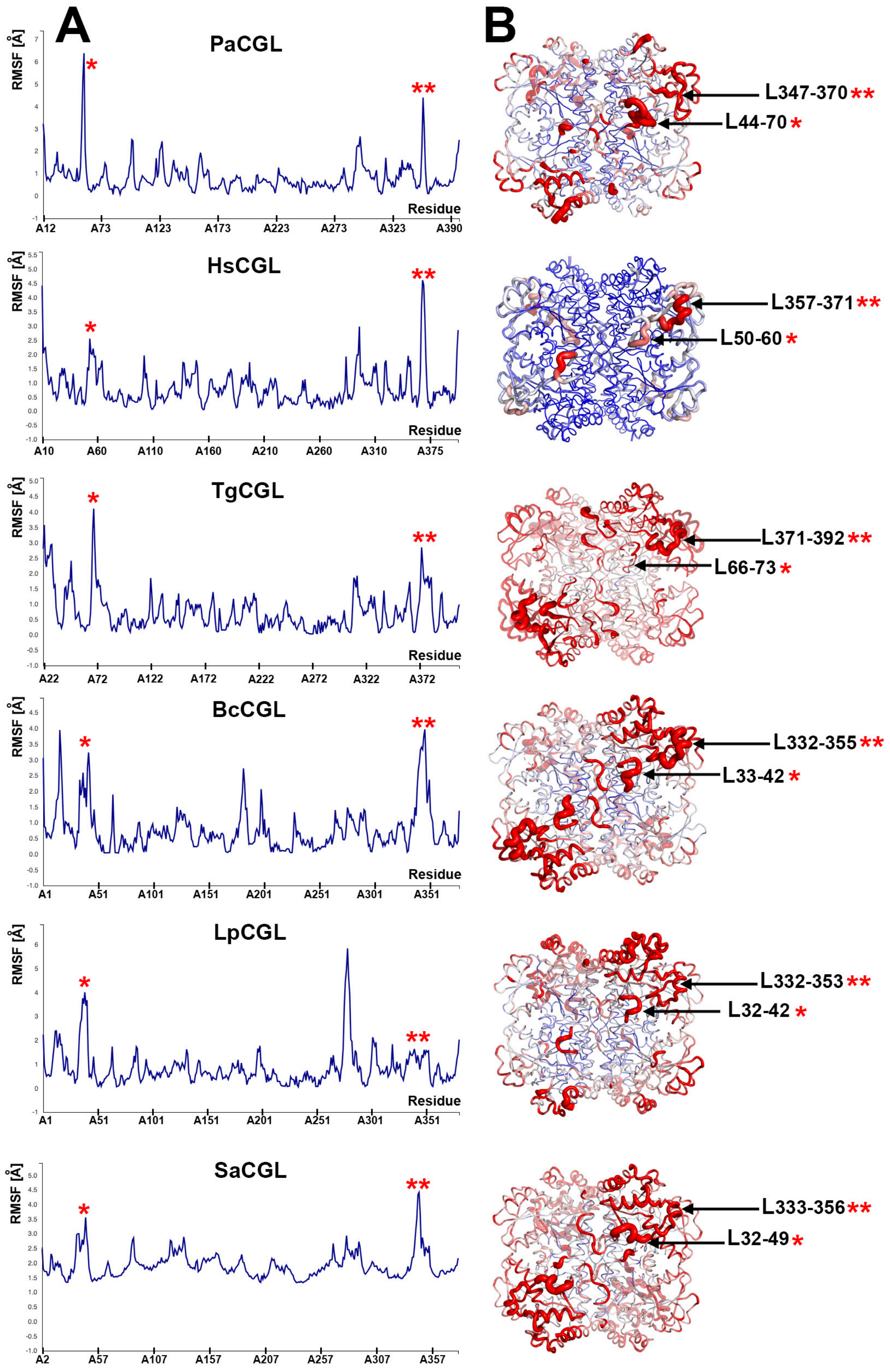

Figure S5. Fluctuation plot analysis (A) and B-thermal parameter (B) distribution found in known CGL structures. (**A**) The structure flexibility of CGLs from different organisms was assessed using the CABSflex tool (https://biocomp.chem.uw.edu.pl/CABSflex2) [27] and the results were presented graphically as Root Mean Square Fluctuation (RMSF) plotted against residues. The regions exhibiting the highest flexibility were denoted by * and **. (**B**) Visualization of B-thermal parameters extracted from the available crystal structures of diverse CGLs; arrows highlight the location of the

flexible loops showing higher B-parameters in the corresponding crystal structures. Interestingly, the highest B-parameter regions match the highest fluctuation segments in the Fluctuation plot analysis, thus indicating the most flexible zones of the CGL proteins. The loops corresponding to regions L23-60 and L347-370 in *Pa*CGL and their homologs exhibit the highest levels of flexibility and the highest B-thermal parameter values. The represented CGLs are as follows: *Pseudomonas aeruginosa* (*Pa*) - PDB code: 7BA4; *Homo sapiens* (*Hs*) - PDB code: 2NMP; *Bacillus cereus* (*Bc*) - PDB code: 7D7O; *Lactobacillus plantarum* (*Lp*) - PDB code: 6LE4; *Toxoplasma gondii* (*Tg*) - PDB code: 8BIZ; *Staphylococcus aureus* (*Sa*) - PDB code: 7MCL.

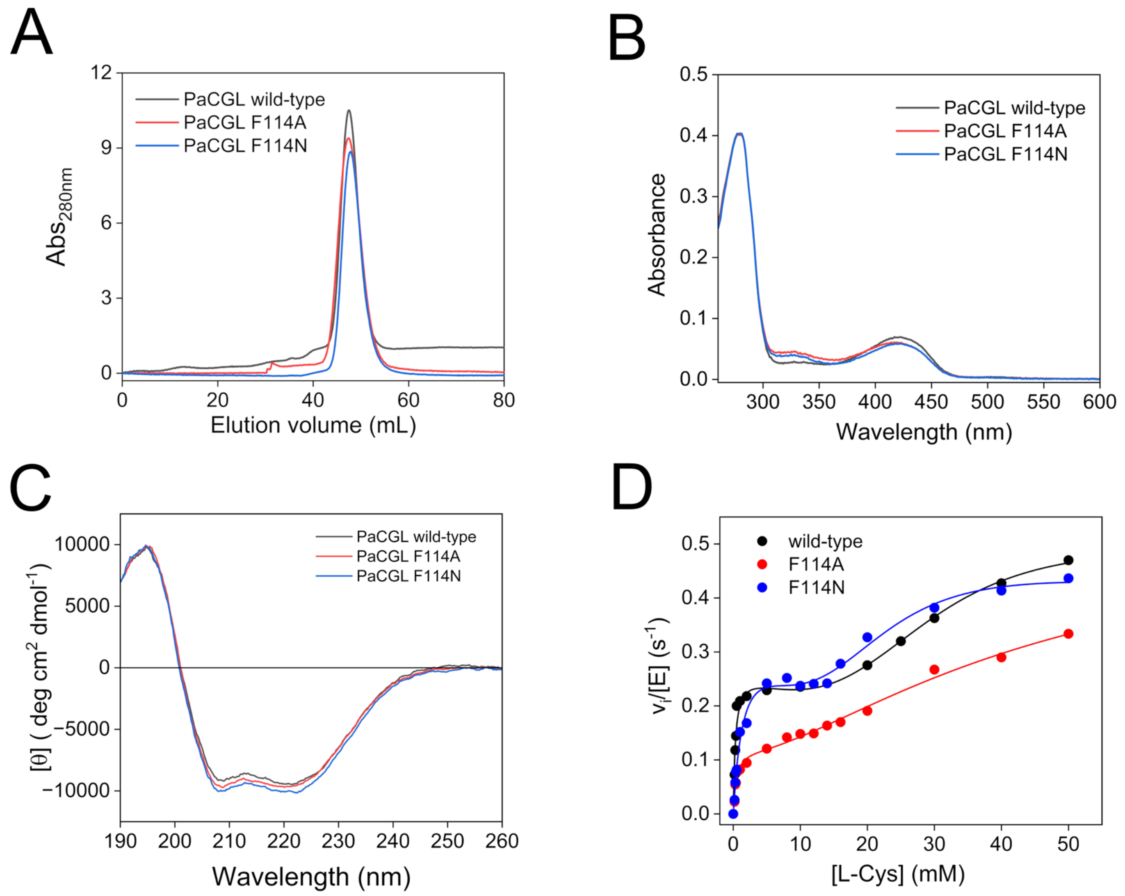

Figure S6. Structural and kinetic properties of *Pa*CGL variants. (**A**) Size exclusion chromatography of *Pa*CGL variants at 2 mg/mL using Sephacryl S-200 16/60 high resolution column. (**B**) UV-visible absorption spectra of 15 µM *Pa*CGL variants recorded in 20 mM sodium phosphate buffer pH 8.0. (**C**) Far-UV CD spectra of 0.2 mg/mL *Pa*CGL variants in 20 mM sodium phosphate buffer pH 8.0. (**D**) Representative kinetics of H_2S generation (**Fig. 1B** main text, reactions **3** + **4**) by *Pa*CGL variants in the presence of L-Cys.

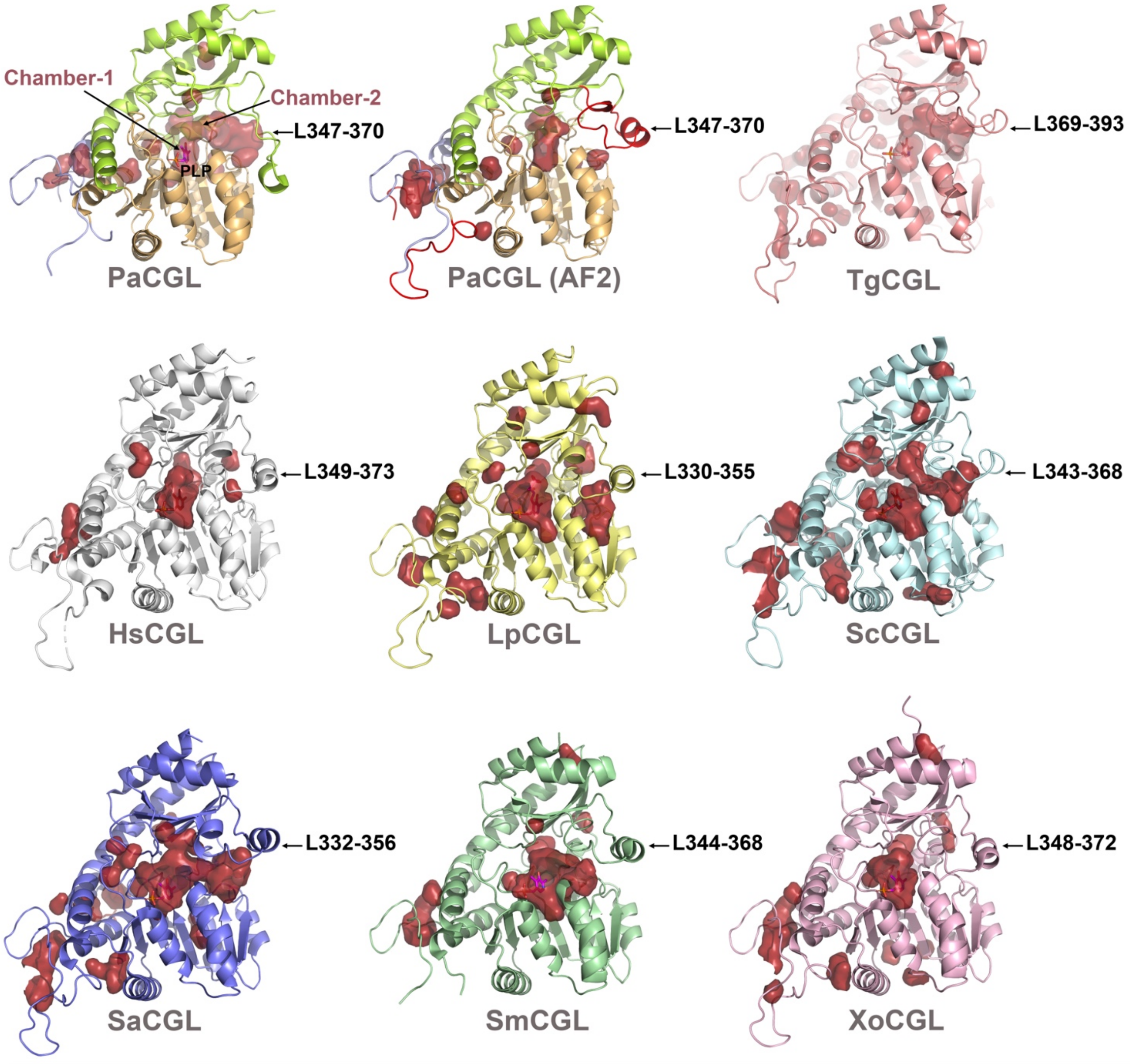

Figure S7. Main cavities found in CGLs. Surface representation of main interior cavities found in all CGLs whose crystal structures are available at present. Chamber-1 corresponds to the catalytic cavity containing the PLP cofactor (in sticks). Chamber-2 is known to host NL1, NL2 and NL3 inhibitors in SaCGL [4]. Interestingly, the size of chamber-1 is essentially conserved in all CGL enzymes. In contrast, chamber-2 varies significantly among species, being smallest in *Hs*CGL, *Xo*CGL or LpCGL. *Pa*CGL-AF2 corresponds to the 3D-model predicted by AF2, where the chamber-2 is significantly smaller and less accessible compared to the chamber-2 identified in our *Pa*CGL crystal structure. Abbreviations: *Pa*CGL, *Pseudomonas aeruginosa*-crystal structure (PDB ID 7BA4); *Pa*CGL-AF2 (*Pseudomonas aeruginosa*, predicted with AF2); *Tg*CGL, *Toxoplasma gondii* (PDB ID 7NL1); *Hs*CGL, Homo sapiens (PDB ID 2NMP); *Lp*CGL, *Lactobacillus plantarum* (PDB ID 6LDO); *Sc*CGL, *Saccharomyces cerevisiae* (PDB ID 1N8P); *Sa*CGL, *Staphylococcus aureus* (PDB ID 7MCB); *Sm*CGL, *Stenotrophomonas maltophila* (PDB ID 6K1L); *Xo*CGL, *Xanthomonas oryzae* (PDB ID 4IYO);

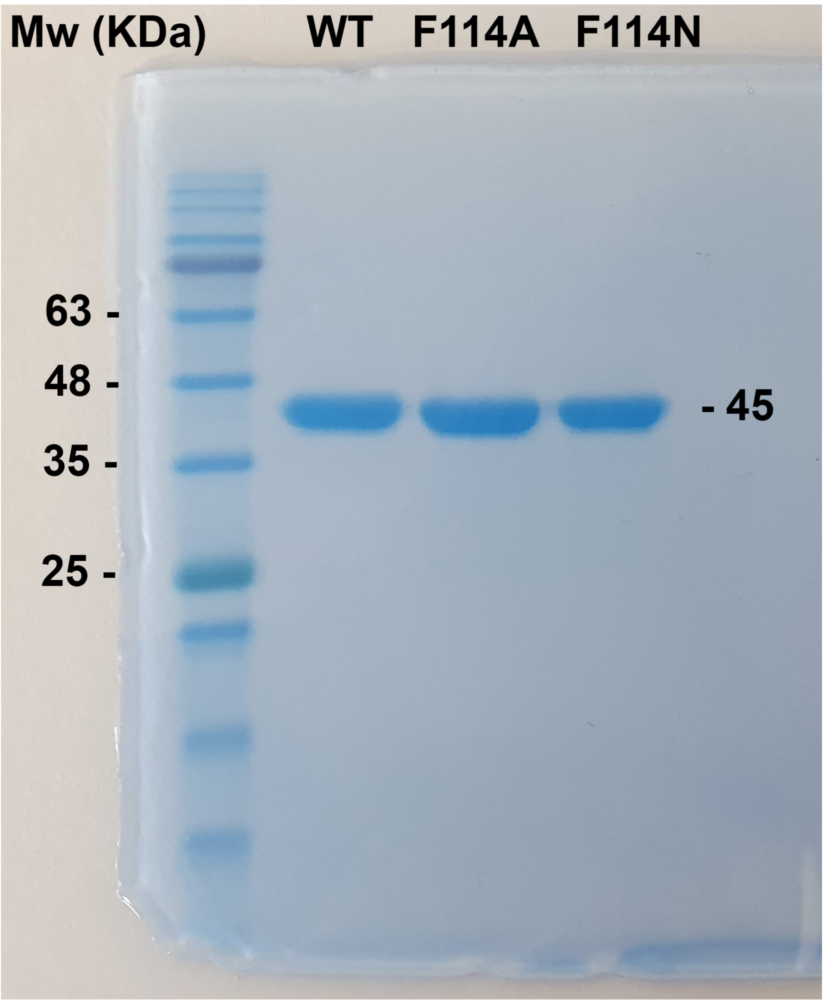

Figure S8. Original uncropped SDS-PAGE gel (12%) of purified recombinant *Pa*CGL variants. The lane on the left corresponds to the protein marker. The Mw of the wt-PaCGL monomer is indicated on the right.

Movie S1. Conformational change of loop L347-370. Movie showing the potential conformational change suffered by loop L347-370 from the extended state found in the crystals to the helical arrangement predicted by Alphafold2 for this loop. The increased helicity of loop L347-370 in the model predicted by Alphafold2 would correspond to a closed conformation of chamber-2. In this state, this cavity significantly restricts its accessibility and internal volume. On the contrary, chamber-2 is more accessible and ready to host small molecules when the loop adopts the extended conformation found in the crystals.
