# Supplementary material for: Catalytic specificity and crystal structure of cystathionine γ-lyase from Pseudomonas aeruginosa
Source: Sci Rep. 2024 Apr 23;14:9364. doi: 10.1038/s41598-024-57625-7 (PMC11039470; doi:10.1038/s41598-024-57625-7)
Supplement: Supplementary file 2 — Supplementary Legends. [file 41598_2024_57625_MOESM2_ESM.pdf]

SUPPLEMENTARY MOVIE LEGEND

Catalytic specificity and crystal structure of cystathionine γ-lyase from *Pseudomonas aeruginosa*

Marco Pedretti[1, #], Carmen Fernández-Rodríguez[2, #], Carolina Conter[1,2], Iker Oyenarte[2], Filippo Favretto[1], Adele di Matteo[3], Paola Dominici[1], Maria Petrosino[4], Maria Luz Martinez-Chantar[2,5], Tomas Majtan[4], Alessandra Astegno[1, *] and Luis Alfonso Martínez-Cruz[2, *]

[1]*Department of Biotechnology, University of Verona, Strada Le Grazie 15, 37134 Verona, Italy.*
[2]*Center for Cooperative Research in Biosciences (CIC bioGUNE), Basque Research and Technology Alliance (BRTA), Bizkaia Technology Park, Building 801A, 48160 Derio, Spain*
[3] CNR Institute of Molecular Biology and Pathology, P.le Aldo Moro 5, 00185 Rome, Italy
[4]*University of Fribourg, Department of Pharmacology, Faculty of Science and Medicine, Chemin du Musee 18, Bldg. PER17, CH-1700 Fribourg, FR, Switzerland.*
[5]*Centro de Investigación Biomédica en Red de Enfermedades Hepáticas y Digestivas (CIBERehd)*

[#] These authors contributed equally to this work.
*Corresponding authors. Address: Luis Alfonso Martínez-Cruz: email: amartinez@cicbiogune.es; Tel: +34 944061318, fax: +34 944-061-301. Alessandra Astegno: email: alessandra.astegno@univr.it; Tel: +39 045-8027955, fax: +39 045-8027929

Supplementary Movie S1. Conformational change of loop L347-370. Movie showing the potential conformational change suffered by loop L347-370 from the extended state found in the crystals to the helical arrangement predicted by Alphafold2 for this loop. The increased helicity of loop L347-370 in the model predicted by Alphafold2 would correspond to a closed conformation of chamber-2. In this state, this cavity significantly restricts its accessibility and internal volume. On the contrary, chamber-2 is more accessible and ready to host small molecules when the loop adopts the extended conformation found in the crystals.
